# A Combination of Culture Conditions and Gene Expression Analysis Can Be Used to Investigate and Predict hES Cell Differentiation Potential towards Male Gonadal Cells

**DOI:** 10.1371/journal.pone.0144029

**Published:** 2015-12-02

**Authors:** Kristín Rós Kjartansdóttir, Ahmed Reda, Sarita Panula, Kelly Day, Kjell Hultenby, Olle Söder, Outi Hovatta, Jan-Bernd Stukenborg

**Affiliations:** 1 Department of Women’s and Children’s Health, Pediatric Endocrinology Unit, Q2:08, Karolinska Institutet and University Hospital, SE-171 76, Stockholm, Sweden; 2 Department of Clinical Science, Intervention and Technology, Division of Obstetrics and Gynecology, Karolinska Institutet, SE-141 86, Huddinge, Sweden; 3 Laboratory of Reproductive Biology, Scientific Unit, Horsens Hospital, DK-8700, Horsens, Denmark; 4 Division of Clinical Research Centre, Department of Laboratory Medicine, Karolinska Institutet, SE-141 86, Huddinge, Sweden; University of Maryland, UNITED STATES

## Abstract

Human embryonic stem cell differentiation towards various cell types belonging to ecto-, endo- and mesodermal cell lineages has been demonstrated, with high efficiency rates using standardized differentiation protocols. However, germ cell differentiation from human embryonic stem cells has been very inefficient so far. Even though the influence of various growth factors has been evaluated, the gene expression of different cell lines in relation to their differentiation potential has not yet been extensively examined. In this study, the potential of three male human embryonic stem cell lines to differentiate towards male gonadal cells was explored by analysing their gene expression profiles. The human embryonic stem cell lines were cultured for 14 days as monolayers on supporting human foreskin fibroblasts or as spheres in suspension, and were differentiated using BMP7, or spontaneous differentiation by omitting exogenous FGF2. TLDA analysis revealed that in the undifferentiated state, these cell lines have diverse mRNA profiles and exhibit significantly different potentials for differentiation towards the cell types present in the male gonads. This potential was associated with important factors directing the fate of the male primordial germ cells *in vivo* to form gonocytes, such as *SOX17* or genes involved in the NODAL/ACTIVIN pathway, for example. Stimulation with BMP7 in suspension culture resulted in up-regulation of cytoplasmic SOX9 protein expression in all three lines. The observation that human embryonic stem cells differentiate towards germ and somatic cells after spontaneous and BMP7-induced stimulation in suspension emphasizes the important role of somatic cells in germ cell differentiation *in vitro*.

## Introduction

Germ cells play the unique role of passing genetic information from one generation to the next. Embryonic cells are partially committed to becoming primordial germ cells (PGCs) as early as at four weeks of gestation [1]. In mice, PGCs originate from the proximal epiblast in the yolk sac in response to stimulation by bone morphogenetic protein (BMP) produced by the nearby visceral endoderm and extra-embryonic ectoderm [2–6]. However, it has not yet been established whether this is also the case in humans, although studies focusing on differentiation of human embryonic stem (hES) cells and male germ cells have demonstrated the importance of BMP4, 7 and 8b for differentiation towards male germ cell development [7–9]. Accordingly, hES cells and human induced pluripotent stem (hiPS) cells appear to be the most promising model systems available for investigations on human germ cell development [10–15]. In earlier studies, pluripotent stem cells from both mice and humans have been differentiated towards the germ cell lineage in adherent cultures on feeder-cell monolayers or on extracellular matrices [9,12,16–19] or as embryoid bodies (EBs) [7,10,20,21] either spontaneously [10,16] or after stimulation with various factors, including BMPs (BMP4, 7, 8b) and all-trans retinoic acid. Although various growth factors and pathways have been evaluated in this context, endogenous factors expressed by the cell lines themselves have not been examined in detail [22–24]. *In vivo* and *in vitro*, male germ cell differentiation is dependent on a well-functioning microenvironment that is present in the testes [25,26]. In that sense, interactions with functional Sertoli and Leydig cells that secrete essential factors and provide structural support are of importance. Detailed studies describing the generation of such a microenvironment derived from hES cells are still missing.

Human ES cell lines do not have identical expression profiles [27] and different hES cell lines exhibit different potentials to differentiate towards the germ cell lineage [28]. The ability to predict this potential would greatly enhance the likelihood of successfully achieving complete spermatogenesis from these cells.

We explored the potential of three male hES cell lines (HS207, HS360 and HS401) to differentiate towards male gonadal cells, by characterizing gene expression both in the undifferentiated state and after differentiation (spontaneously or in response to BMP7) for 14 days on feeder cells (human foreskin fibroblasts (hFFs)) or in suspension culture [29] without any supporting feeder cells. Subsequent analyses of the morphology, mRNA and protein profile, and functions of the differentiated cells, revealed that each of these three cell lines exhibits a different potential to differentiate towards the male germ line or into gonadal somatic cells. In this study, we demonstrated for the first time that the analysis of gene expression profiles from two different culture conditions can be used to predict the potential of cell lines to differentiate towards the male germ cell lineage and male gonadal somatic cells.

## Materials and Methods

### Ethics approval

Approval for the use of human cells and tissue samples was obtained from the Ethics Board of Karolinska Institutet and the Regional Ethics Board in Stockholm. All patients gave written informed consent for donating samples and studies were performed according to the amended Declaration of Helsinki. The hES cell lines HS207 (XY), HS360 (XY) and HS401 (XY) were derived earlier at Karolinska Institutet (Karolinska University Hospital Huddinge, Stockholm, Sweden) [30–32].

### Human embryonic stem cell cultures

Adherent cultures of hES cells were obtained on mitotically inactivated hFFs (CRL-2429; ATCC, VA, USA) in Knockout Dulbecco´s Modified Eagle´s medium (KO-DMEM, 10829018, Life Technologies, CA, USA) supplemented (20%) with KO Serum Replacement (KO-SR, 10828028, Life Technologies), 0.5% penicillin-streptomycin (Pen/Strep, 15140122, Life Technologies), 2 mM L-GlutaMAX (35050038, Life Technologies), 1% non-essential amino acids (NEAA, 11140035, Life Technologies), 0.5 mM 2-mercaptoethanol (31350010, Life Technologies) and 8 ng rFGF2/ml (234-FSE/CF, R&D Systems, MN, USA) as described previously [10,28,32]. The medium was changed 6 times each week and the cells passaged mechanically at intervals of 4–6 days.

To obtain spheres in suspension culture, hES cell colonies were scraped off the hFF layer with a scalpel and placed (without hFFs) in 24-well ultra-low-attachment-surface plates (3473, Corning Inc., NY, USA) in Neurobasal medium (21103049, Life Technologies) supplemented (14%) with KO-SR, 2 mM GlutaMAX, 0.5% Pen/Strep, 1% NEAA, 20 ng rFGF2/ml, 25 ng rActivin A/ml (120-14E, Peprotech, NJ, USA), 1.0 μg human fibronectin/ml (354008, BD Biosciences, NJ, USA), 0.5 μg human placental laminin/ml (L6274, Sigma Aldrich, MO, USA), 0.001% porcine gelatin (G2625, Sigma Aldrich), 10 ng each of recombinant brain-derived neurotrophic factor (rBDNF, 450–02, Peprotech), recombinant neurotrophin-3 (rNT3, 450–03, Peprotech) and recombinant neurotrophin-4 (rNT4, 450–04, Peprotech) per ml, and 1 × Nutridoma (11011375001, Roche, Basel, Switzerland), as described previously [29]. The medium was changed every other day and the spheres were passaged mechanically at intervals of 8–10 days.

### Differentiation of human embryonic stem cells

Adherent-culture differentiation of hES cells took place for 14 days on hFFs in normal medium without rFGF2, in the presence and absence of 10 ng BMP7/ml (354-BP-010, R&D Systems). During differentiation the cells were not passaged and the medium was changed every other day.

Differentiation in suspension took place for 14 days, either in normal non-stimulated conditions in 50% KO-DMEM (10829018, Life Technologies) and 50% F12 (21127, Life Technologies) or with stimulation by adding 10 ng BMP7/ml. During differentiation the spheres were not passaged and the medium was changed every other day. Following this 14-day differentiation, the cells were stimulated with 5 IU each of human chorionic gonadotrophin (hCG, Pregnyl 5000IE, Merck Sharpe & Dohme, USA) and recombinant follicle-stimulating hormone (rFSH, Gonal F 75IE, Merck, Germany) per ml for 48 hours.

### RNA extraction and cDNA amplification

Total RNA was isolated with TRIzol reagent (15596–018, Life Technologies) in accordance with the manufacturer’s protocol and subsequently treated with DNase 1 (AMPD1, Sigma Aldrich). One μg total RNA was reverse-transcribed by using an IScript cDNA Synthesis Kit (170–8891, Bio-Rad, CA, USA) in accordance with the manufacturer’s protocol.

### Validation of endogenous controls

We validated six endogenous controls for our expression analyses *(GAPDH*, *ACTB*, *RAF1*, *CTNNB1*, *EEF1A1* and *18S)*. These endogenous controls have been suggested to be the most stable ones when analysing gene expression in hES cells [27]. We found the first three *(GAPDH*, *ACTB*, *RAF1)* to be most stable in our analyses, and, hence, chose to use them for our expression analyses.

### Reverse transcriptase-polymerase chain reactions (RT-PCRs)

RT-PCR analysis was performed on a 2710 Thermal Cycler (Life Technologies, Carlsbad, CA, USA), using the Expand High Fidelity PCR System (11759078001, Roche) with primers specific for messenger RNAs considered to be consensus markers for undifferentiated hES cells (S1 Table) and *ACTB* as an endogenous control.

### Quantitative PCRs (Q-PCRs)

Q-PCR analysis was performed on an iCycler iQ multicolor RT PCR detection system (Bio-Rad, Hercules, CA, USA) using TaqMan Gene Expression Master Mix (4369510, Life Technologies) for analysis with TaqMan Gene expression assays (Life Technologies; S2 Table). iQ SYBER^®^ Green Super mix (170–8882, Bio-Rad) was employed for analysis with SYBR Green primers (S3 Table). *GAPDH* was the endogenous control.

The ddCt (delta delta cycle threshold) method was utilized to analyse gene expression in accordance with the recommendations from Life Technologies. In brief, the mean of triplicate values for each sample was normalized to the mean value for *GAPDH* in the same sample (dCt). Thereafter, all these values were normalized to a defined standard (ddCT) and gene expression finally expressed as fold-change (2^-ddCT^).

### TaqMan Low-Density Arrays (TLDAs)

TLDA cards (4385344, Life Technologies) for human stem cell pluripotency were used to compare the three undifferentiated hES cell lines (HS207, HS360 and HS401) cultured on supporting hFFs or as spheres in suspension. These cards, designed for the International Stem Cell Initiative [27] and based on TaqMan chemistry, are used to quantify the expression of 90 relevant and six control genes. Each cell line was analysed in triplicate under both culture conditions, except for HS401 in suspension, where, for technical reasons, only one analysis could be performed. Samples without any expression were assigned a value equal to the highest dCt+1 (i.e., 18.0755). This was subtracted from the other values in order to scale the data so that high values reflect high expression and zero equals no expression. Mean values of the replicates were used for heat maps and clustering analysis (Euclidean distance with complete linkage) using GENE-E software (http://www.broadinstitute.org/cancer/software/GENE-E/index.html).

### Morphological evaluation of hES cells

The hES cells, as well as control testicular biopsy samples from a one-year-old boy and a man were photographed under a Nikon SMZ-U microscope (Nikon, Shinjuku, Tokyo, Japan) with an Infinity 1 camera (Lumen*era* corporation, Ottawa, Ontario, Canada) (S1 Fig). In brief, for this purpose the samples were fixed in 4% paraformaldehyde (PFA) overnight at 4°C, dehydrated with gradually increasing concentrations of aqueous ethanol, embedded in paraffin (P3808, Sigma Aldrich) and cut into 4–5 μm-thick sections for staining with Periodic acid/Schiff’s reagent (PAS, 1.01644, Merck, Germany). Morphology was examined microscopically (Eclipse E800; Nikon, Shinjuku, Tokyo, Japan) and photographs taken with a 12.5 million-pixel cooled digital colour camera (Olympus DP70, Shinjuku, Tokyo, Japan). The different cell types were identified on the basis of size, shape and location, according to Russel and colleagues [33].

### Transmission electron microscopy (TEM)

TEM was performed as previously described by Ruzzenente and co-workers [34]. In brief, the cells were first fixed in 2.5% glutaraldehyde in 0.1 M phosphate buffer, pH 7.4, in a refrigerator, then rinsed with the same phosphate buffer and post-fixed in 2% osmium tetroxide in this buffer at 4°C for 2 hours, dehydrated in ethanol and then acetone and embedded in LX-112 (Ladd, Burlington, Vermont, USA). Ultrathin sections (approximately 40–50 nm thick) were prepared with a Leica ultracut UCT (Leica; Wien, Austria), contrasted with uranyl acetate and then lead citrate and examined under a Tecnai 12 Spirit Bio TWIN transmission electron microscope (FEI Company, Eindhoven, Netherlands) at 100 kV. Digital images were captured using a Veleta camera (Olympus Soft Imaging Solutions, GmbH, Münster, Germany) and the different cell types identified on the basis of size, shape and location according to the criteria formulated by Sathananthan and colleagues [35].

### Immunohistochemical and immunofluorescence staining

For this purpose, spheres of cells and the control samples (see above) were fixed in 4% PFA overnight at 4°C, dehydrated with gradually increasing concentrations of ethanol and thereafter n-butyl acetate (442666-U, Sigma Aldrich), embedded in paraffin (P3808, Sigma Aldrich), cut into 4–5 μm sections and placed on glass slides (J1800AMNZ, Gerhard Menzel, Germany). Following rehydration with xylene and gradually decreasing concentrations of aqueous ethanol, the samples for immunohistochemical (IHC) staining were blocked with 10% serum (horse or goat, depending on the secondary antibody applied) in phosphate-buffered saline (PBS) with 0.1% bovine serum albumin (BSA, 011-000-162, Jackson Immunoresearch, USA) for 20 min. Samples for immunofluorescence (IF) staining were first subjected to antigen retrieval using citrate buffer (pH 6.0) at 95°C for 30 min and cooled down for another 30 min, followed by peroxidase blocking using 3% H_2_O_2_ in methanol for 30 min at room temperature (RT) and afterwards incubated for 30 min in 20% chicken serum (C5405, Sigma Aldrich, Germany), or 10% donkey serum (017-000-001, Jackson Immunoresearch, USA), 5% BSA-1×Tris-buffered saline (TBS). After incubation with primary antibodies or unspecific IgGs (used as negative controls at the same concentration) (S4 Table) dissolved in 0.1% BSA-1× PBS (for IHC staining) or 20% chicken serum, 5% BSA-1× TBS (for IF staining) overnight at 4°C, the slides were washed three times for 5 min each with 1× PBS (for IHC staining) or with 1× TBS (for IF staining).

Following subsequent incubation with secondary antibodies specific for the primary antibodies applied (S5 Table), again in 0.1% BSA-1× PBS, for 1 hour at 37°C, the slides were washed three times for 5 min each with 1× PBS, incubated with ABC reagent from a Vectastain ABC kit (PK6100, Vector Laboratories, CA, USA) for 30 min at 37°C, washed again three times and thereafter stained with DAB for 30–60 sec at RT. Finally, the slides were washed twice for two min each in H_2_O, incubated for 5 sec in Hämalaun solution (1.09249.1000 Merck), rinsed for 5 min with tap water, dehydrated with gradually increasing concentrations of aqueous ethanol and then xylene, and mounted.

For the IF staining, after washing with 1X TBS the slides were incubated either with a secondary antibody conjugated to Cy3 or with a secondary antibody conjugated to HRP depending on the type of staining. The secondary antibodies were diluted in blocking serum and the incubation was for 30 min at RT. After washing, the samples were incubated with TSA-Plus Fluorescein (NEL741001KT, Perkin Elmer, USA) or TSA-Plus Cy3 (NEL744001KT, Perkin Elmer) according to the manufacturer’s protocol. After washing, the samples were mounted in Vectashield mounting medium with Dapi (H-1500, Vectro, USA). All stained sections were photographed under a microscope (Eclipse E800; Nikon; Japan) with a 12.5 million-pixel, cooled digital colour camera (Olympus DP70, Japan). Evaluation of DDX4- and SOX9-expressing cell colonies was carried out by manual examination of at least 45 cell colonies from each cell line (experiments run in triplicate and pooled for examination).

### Enzyme-linked immunoassay (ELISA) of Inhibin B Gen II

Media collected from cell cultures stimulated with rFSH and/or hCG for 48 hours were stored at -80°C until analysis with ELISA kits for Inhibin B Gen II (A81303, Beckman Coulter, CA, USA) utilizing calibrators and controls (A81304, Beckman Coulter) in accordance with the manufacturer’s recommendations.

### Assay of testosterone

By adding 0.5 ml ethyl acetate (300612, Merck), followed by 15 min of shaking, testosterone was extracted from media collected after stimulation with rFSH and/or hCG for 48 hours. Two-min centrifugation at maximal speed provided a supernatant and a lower phase that was then re-extracted in the same manner. The combined ethyl acetate extracts were evaporated overnight and the resulting pellets dissolved in 1× PBS and assayed for testosterone by using COAT-A-COUNT^®^ kits (TKTT2, Siemens, Munich, Germany) in accordance with the manufacturer´s protocol. In brief, the extracted samples were incubated with added I^125^ testosterone at 37°C for 3 hours, the solution then decanted and the radioactivity in the tube counted in a Gamma counter (1470 Wizard Wallac, GMI, Ramsey, MN, USA) for one min. For calibration, standards provided with the kits (0–55 nmol/l) were employed.

### Statistical analysis

For comparing two different culture conditions, hFF (n = 9) and suspension (n = 7) when using TLDA arrays, the unpaired t-test was used with a two-tailed p value (Prism 6, GraphPad Software, Inc., CA, USA). For comparing all cell lines and culture conditions separately, one-way ANOVA was used with the Bonferroni multiple comparison test (Prism 6, GraphPad Software, Inc., CA, USA). Levels of gene expression are expressed as means ± standard deviations (SDs). One-Way RM ANOVA (SigmaPlot 11.0; Systat Software Inc., CA, USA) was used to compare values under the different experimental conditions. Following the Shapiro–Wilk test for normality, pair-wise multiple comparisons were performed with Holm–Sidak or Dunnett’s procedure (SigmaPlot 11.0; Systat Software Inc.; CA, USA), as indicated in the figure legends. A p-value of ≤ 0.05 was considered statistically significant.

## Results

### Undifferentiated hES cells demonstrate cell line-specific patterns of gene expression when cultured on hFFs or in suspension

**Fig 1:**
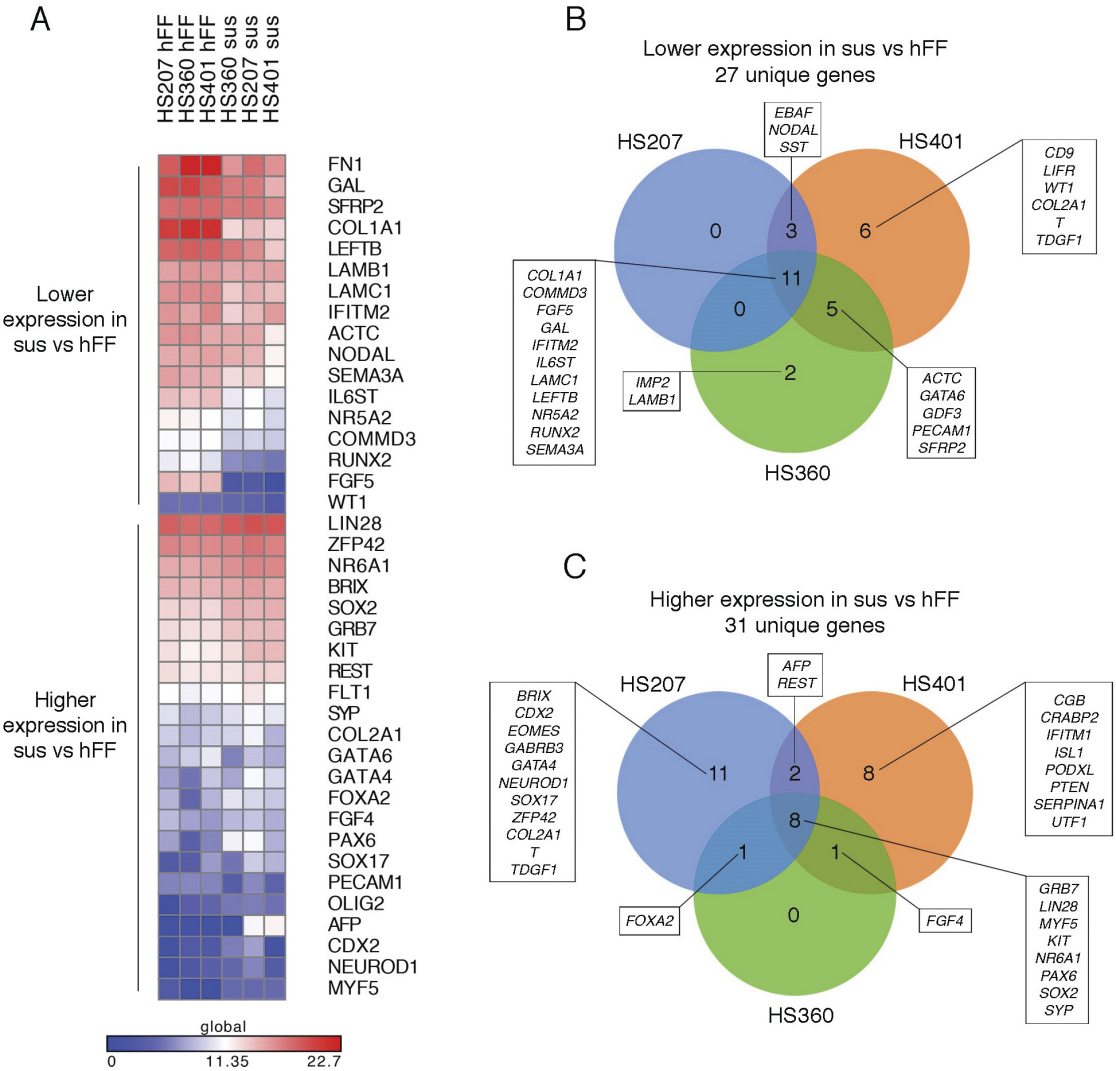
TaqMan Low-Density Array analysis of undifferentiated hES cells cultured on hFFs (hFF) or in suspension (sus). (A) Significantly lower and higher (p < 0.05) expression of genes in suspension culture relative to hFF culture. The expression values are dCt values relative to the mean expression of *ACTB*, *GAPDH* and *RAF1*, scaled by subtracting the dCt value from the lowest dCt+1 value. The value of dCt+1 was assigned to samples with no expression; thus blue (0) represents no expression, with increasing expression towards red. (B) Cell line-specific differences in gene expression between hFF and suspension culture conditions, with lower expression in suspension versus hFF cultures, and (C) higher expression in suspension versus hFF cultures (p < 0.05 for HS207 and HS360 cells). A list of gene names and abbreviations can be found in S6 Table.

We cultured three hES cell lines (HS207, HS360, and HS401) on mitotically inactivated hFFs or in pluripotency-supporting suspension cultures. The cells grew in typical hES cell colonies or in compact spheres, respectively (shown for HS360 in S1A–S1C Fig), and expressed the consensus markers for pluripotency in both culture conditions (S2 Fig). Several genes (including *POU5F1*, *NANOG* and *TERT*) were expressed at similar levels in the two culture conditions, when analysed by the TaqMan Low-Density Array (TLDA) cards with 96 different TaqMan assays designed for pluripotency assessment [27] (S6 Table). The samples clustered in relation to the culture condition rather than the cell line, indicating differences in gene expression profiles between the culture conditions (S7 Table). Indeed, we found 17 genes with significantly lower expression and 23 genes with significantly higher expression in suspension culture than on hFFs (p < 0.05, Fig 1A). Interestingly, expression of the stem cell markers *SOX2*, *KIT*, *FGF4*, *LIN28* and the endodermal marker *SOX17*, all of which are also related to germ cells, were up-regulated in suspension cultures, whereas *FGF5* and *GAL* were down-regulated (Fig 1 and S7 Table). We found some endodermal and mesodermal markers either up- or down-regulated in suspension cultures relative to hFF cultures, indicating no clear differentiation (S7 Table). We did, however, find a few ectoderm-related markers (including *PAX6*, *OLIG2*, and *NEUROD1*) up-regulated in suspension cultures, whereas no ectodermal markers were down-regulated, most likely as a result of the neurotrophic factors used in the suspension medium (Fig 1 and S7 Table). We observed some changes in the NODAL/ACTIVIN signalling pathway in relation to the culture conditions, with down-regulation of *NODAL* and *LEFTB* in suspension cultures relative to hFF cultures (Fig 1 and S7 Table). Other markers related to this pathway were similar in the two culture set-ups when all three cell lines were compared, although some expression differences were observed in individual cell lines.

We next compared the effect of the culture condition in each cell line separately. In addition to the changes in gene expression common to all cell lines, we found cell line-specific differences between culture conditions (Fig 1B and 1C and S8 Table). *EBAF* and *NODAL* expression was lower in suspension cultures of HS207 and HS401 cells relative to hFF culture and *GDF3* expression was lower in HS401 and HS360 cells. *LEFTB* expression was lower in all cell lines in suspension culture conditions. Interestingly, *TDGF1* expression was lower in HS401 cells, but higher in HS207 cells in suspension culture relative to hFF culture, while it remained similar in HS360 cells. The germ cell-related markers *SOX2*, *KIT* and *LIN28* showed higher expression in suspension culture of all cell lines, whereas *FGF4* expression was higher in HS401 and HS360 cells, and *SOX17* expression only in HS207 cells. As regards the markers related to the different germ layers, HS360 showed the most stable gene expression in the two culture conditions. In general, HS360 also showed the most stable gene expression of markers in the NODAL/ACTIVIN signalling pathway, while still showing up-regulation of *SOX2*, *KIT* and *LIN28*, suggesting that HS360 could have higher potential for germ cell differentiation relative to the other lines.

### Absence of rFGF2 from the medium results in down-regulation of pluripotency markers in cultures of hES cells in suspension, but not cells on hFFs

**Fig 2:**
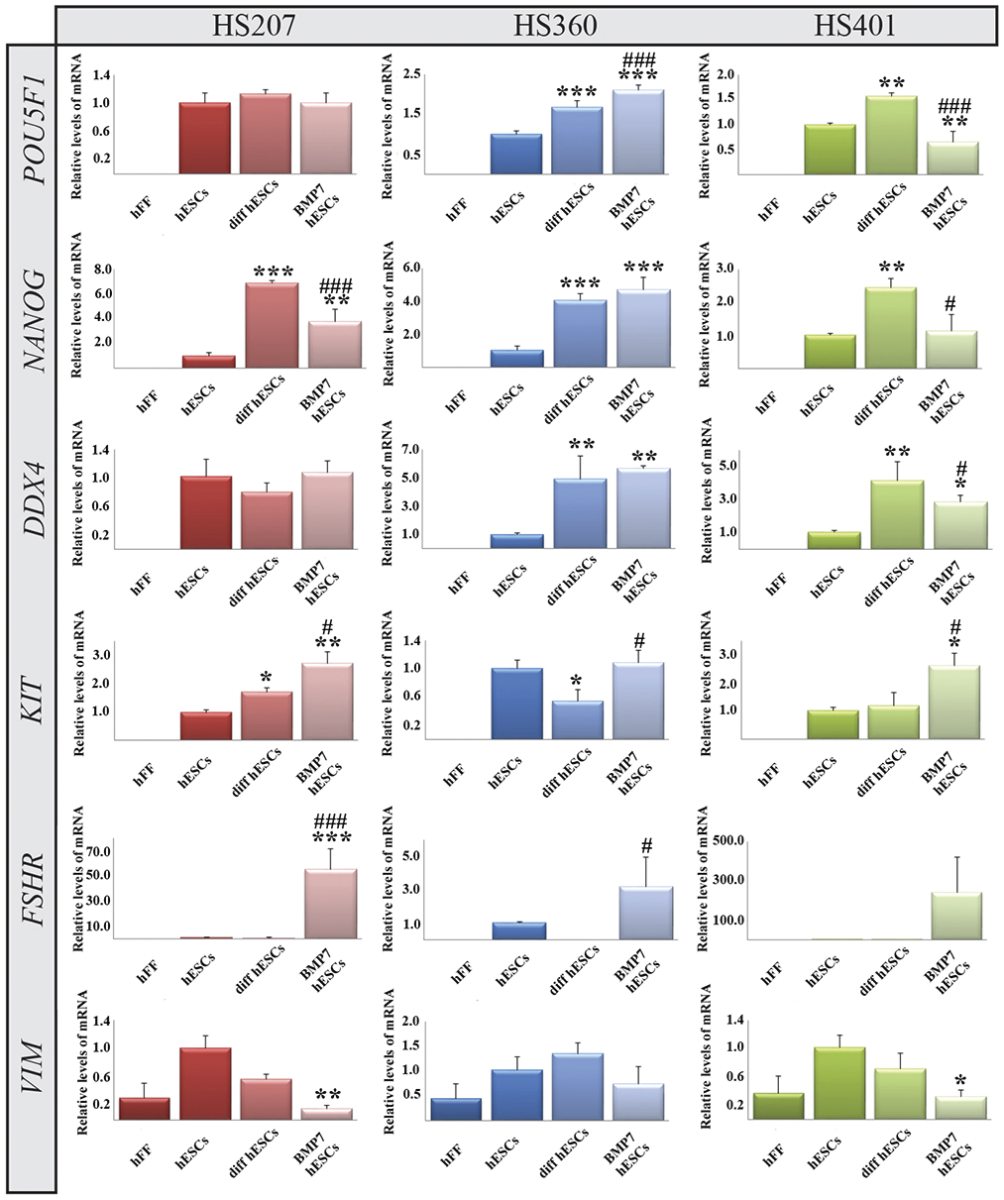
Expression of mRNA encoding the pluripotency markers *NANOG* and *POU5F1*, the germ cell markers *DDX4* and *KIT* and Sertoli cell markers *FSHR* and *VIM* by HS207, HS360 and HS401 cells cultured and differentiated on hFFs. The relative level of mRNA was calculated by the ddCt procedure from the mean of triplicates and statistical analysis performed by way of One-way RM ANOVA. The error bars depict standard deviations. *p <0.05, **p <0.01, ***p <0.001 when comparing undifferentiated with differentiated cells. #p <0.05, ##p <0.01, ###p <0.001 when comparing BMP7-stimulated with unstimulated cells. Abbreviations: hFFs: human foreskin fibroblasts; hESCs: undifferentiated human embryonic stem cells; diff hESCs: spontaneously differentiated hESCs; BMP7 hESCs: hESCs stimulated to differentiate by bone morphogenetic protein 7 (BMP7). A list of gene names and abbreviations can be found in S6 Table.

To evaluate their potential to differentiate towards male germ cells, we allowed the three hES cell lines to differentiate spontaneously on hFFs and in suspension. Spontaneous differentiation on hFFs has been described previously [16,28] and it was therefore used as a basic control for differentiation in suspension. To obtain this spontaneous differentiation on hFFs, the hES cells were cultured for 14 days without I) adding rFGF2 to the media, II) passaging the cells and III) adding any other stimuli, as described previously [28]. Such differentiation on hFFs was associated with similar or, more often, elevated expression of pluripotency markers (*NANOG* and *POU5F1)* by all three cell lines (Fig 2). Thus we conclude that the attempted spontaneous differentiation on hFFs was at least partly ineffective. This emphasizes the supportive role of factors such as FGF2 and activin A, both of which are known to be secreted by hFFs [36–38], on the expression of pluripotency genes. Expression of the germ cell marker *DDX4* was up-regulated in HS360 and HS401 cells, but not HS207 cells (Fig 2). Expression of *KIT*, a marker of both undifferentiated hES cells and early germ cells, was elevated in HS207 cells, down-regulated in HS360 cells and unchanged in the HS401 cell line (Fig 2). Levels of mRNA encoding the Sertoli cell markers *FSHR* and *VIM* were not altered significantly after spontaneous differentiation for two weeks (Fig 2).

**Fig 3:**
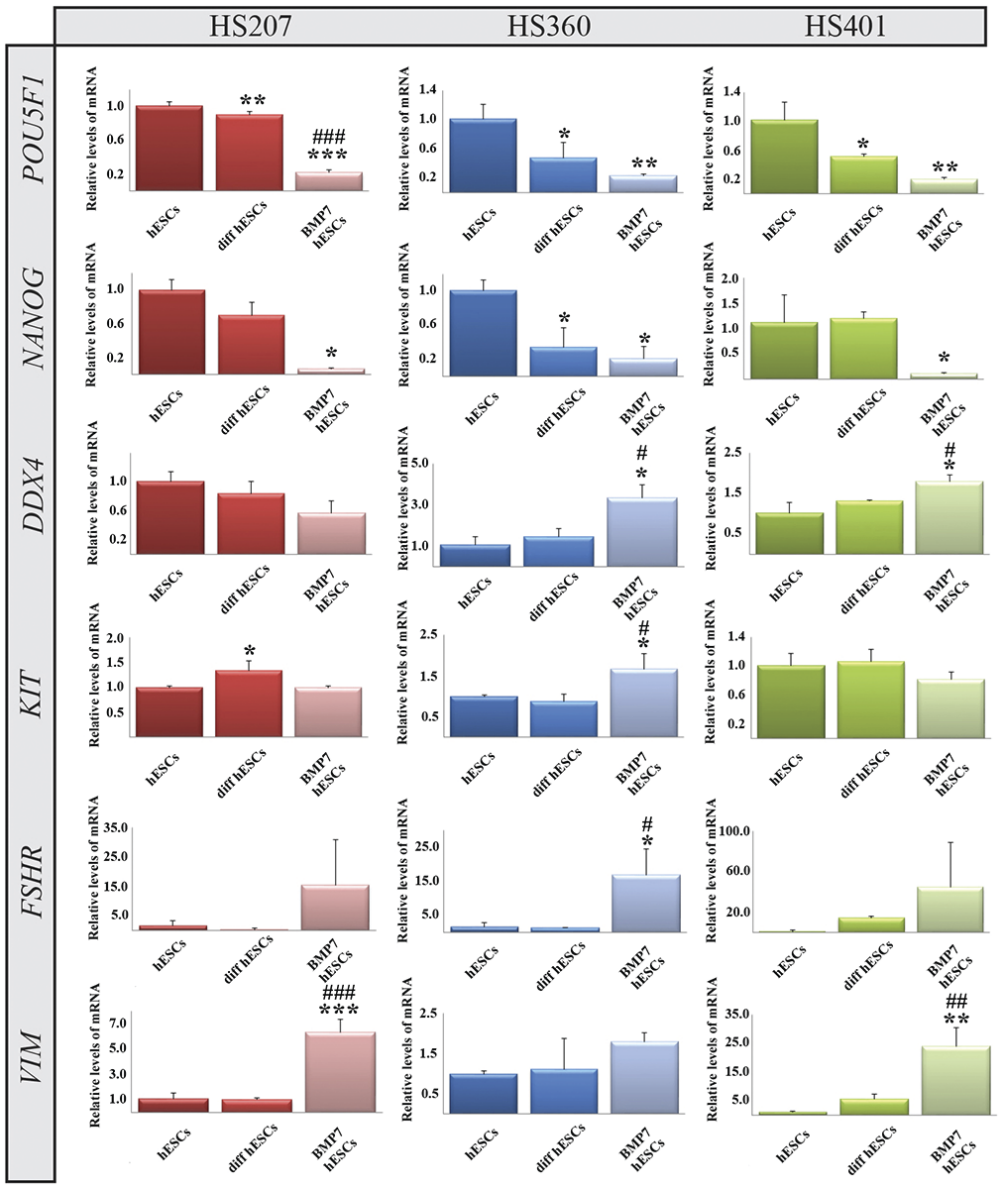
Expression of mRNA encoding the pluripotency markers *NANOG* and *POU5F1*, the germ cell marker *DDX4* and Sertoli cell markers *FSHR* and *VIM* by HS207, HS360 and HS401 cells cultured and differentiated in suspension. The relative level of mRNA was calculated by the ddCt procedure from the mean of triplicates and statistical analysis performed by way of One-way RM ANOVA. The error bars depict standard deviations. *p <0.05, **p <0.01, ***p <0.001 when comparing undifferentiated with differentiated cells. #p <0.05, ##p <0.01, ###p <0.001 when comparing BMP7-stimulated with unstimulated cells. For an explanation of the abbreviations, see the legend to Fig 2.

To achieve spontaneous differentiation in suspension, the cells were kept in un-supplemented suspension medium for two weeks without passaging. In comparison with undifferentiated hES cells cultured in the same manner, the level of *POU5F1* mRNA was significantly down-regulated in all three cell lines while that encoding *NANOG* was significantly down-regulated only in HS360 cells (Fig 3). Expression of *DDX4* was not significantly altered after spontaneous differentiation in any cell line, whereas the level of *KIT* mRNA in HS207 cells was elevated (Fig 3). Levels of mRNA encoding markers of Sertoli (*FSHR* and *VIM*) and Leydig cells (*HS3BD1* and *INSL3*) were low and did not change (Fig 3 and S3A Fig).

Morphological evaluation of hES cell colonies in suspension by transmission electron microscopy revealed different cell types, some exhibiting morphological similarities to undifferentiated embryonic stem cells and others resembling differentiated hES cells (S4A and S4B Fig, respectively).

### BMP7 stimulates differentiation of hES cells in suspension towards male gonadal cells

Differentiation of hES cells on hFFs without adding exogenous rFGF2 to the culture medium, but, instead, supplementing this medium with human recombinant BMP7, resulted in similar or up-regulated expression levels of mRNA encoding the pluripotency markers *NANOG* and *POU5F1* (Fig 2), as observed in the case of spontaneous differentiation. Levels of *DDX4* mRNA were elevated in HS360 and HS401 cells, but unchanged in HS207 cells (Fig 2). *KIT* mRNA was up-regulated in the HS207 and HS401 cells, but not in the HS360 cell line. With respect to Sertoli cell markers, the level of *FSHR* mRNA was elevated significantly in HS207 cells, while *VIM* mRNA was expressed at a lower level in HS207 and HS401 cells (Fig 2).

Following differentiation in suspension medium supplemented with human recombinant BMP7 and without exogenous rFGF2, the levels of mRNA encoding the pluripotency markers *NANOG* and *POU5F1* were significantly lower than the pre-differentiation levels in all three cell lines (Fig 3). The levels of *DDX4* mRNA in HS360 and HS401 cells were significantly higher both in comparison to undifferentiated and spontaneously differentiated cells, as were the levels of mRNA encoding another germ cell marker, *KIT*, in HS360 cells (Fig 3). Evaluation of Sertoli cell markers revealed that *FSHR* mRNA was expressed at higher levels in HS360 cells, while higher expression of *VIM* was observed after BMP7 stimulation in HS207 and HS401 cells. Leydig cell markers (*HS3BD1* and *INSL3*), expressed at very low levels or not at all in undifferentiated cells, were present at significantly higher levels as regards *INSL3* in HS207 cells and *HS3BD1* in HS360 cells following differentiation in suspension with BMP7 stimulation (S3A Fig).

**Fig 4:**
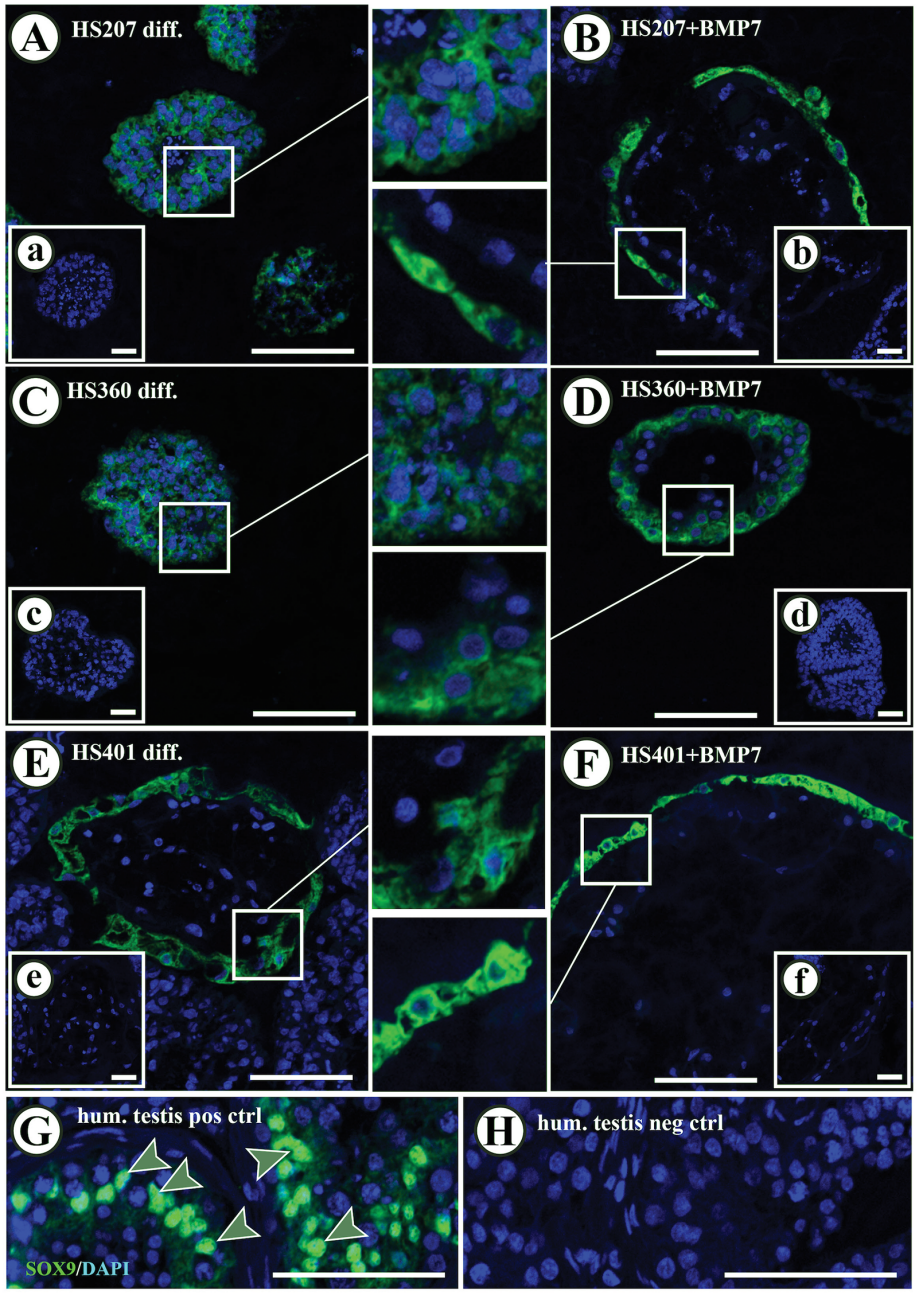
Immunofluorescence detection of SOX9 in HS207, HS360 and HS401 cells cultured in suspension. Cytoplasmic expression of SOX9 in cells on the edges of the cell spheres was observed in all hES cell lines after BMP7-stimulation (B, D and F) as well as after spontaneous differentiation (A, C and E). Nuclear expression of SOX9 was observed in Sertoli cells present in adult human testis (G; arrow heads). Negative controls exhibited no specific SOX9 staining in either the nucleus or cytoplasm (H and small inserts in A to F). Green: SOX9 staining. Blue: DAPI staining marking the nucleus. Scale bars: 100 μm in A to H, and 50 μm in the small inserts.

**Fig 5:**
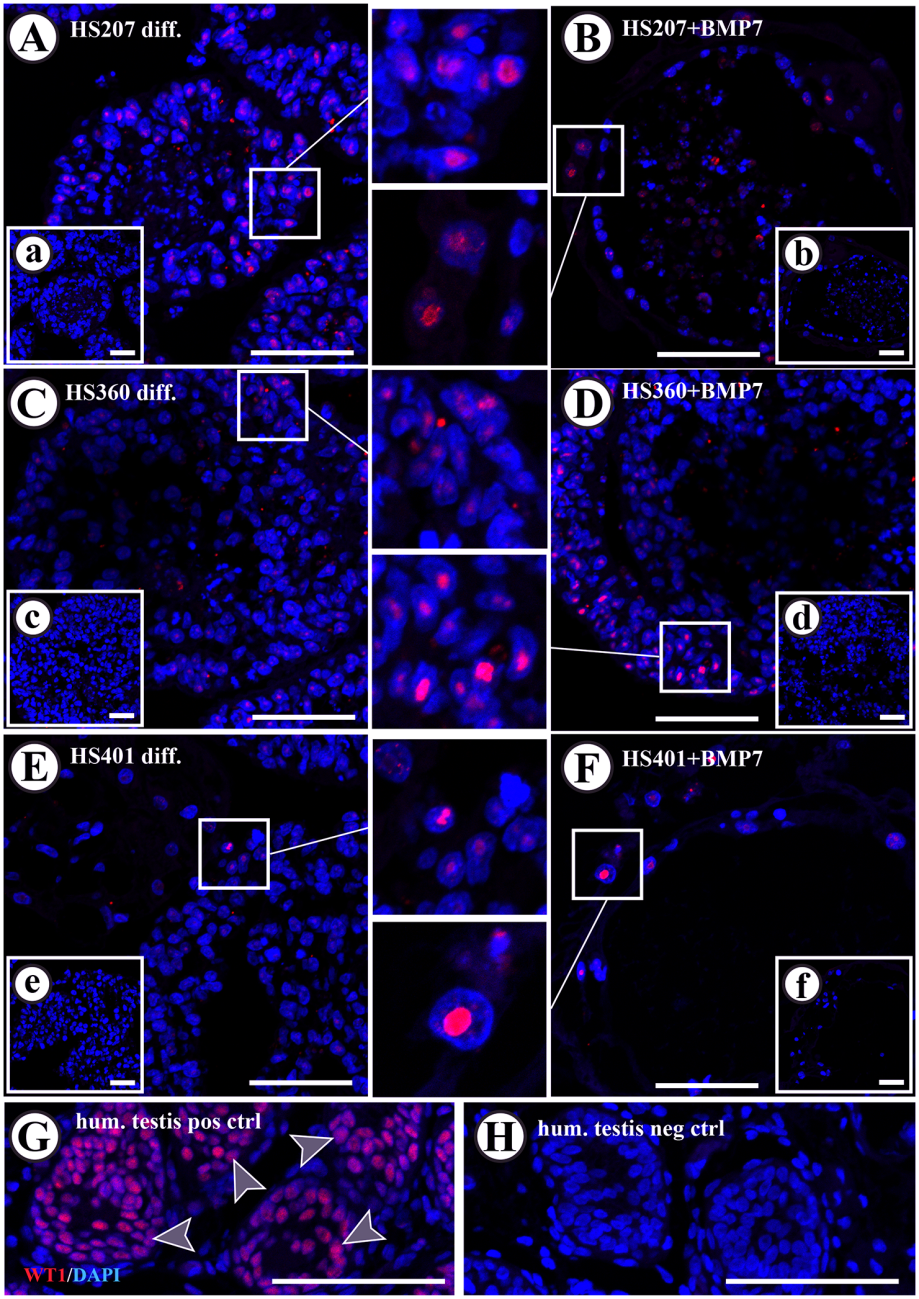
Immunofluorescence detection of WT1 in HS207, HS360 and HS401 cells cultured in suspension. Nuclear expression of WT1 in cells was observed in all hES cell lines after spontaneous differentiation (A, C, and E) as well as after BMP7-stimulation (B, D and F) in cells mainly located on the edge of the spheres. Nuclear expression of WT1 was observed in Sertoli cells present in adult human testis (G; arrow heads). Negative controls exhibited no specific WT1 staining in either the nucleus or cytoplasm (H and small inserts in a-f). Red: WT1 staining. Blue: DAPI staining marking the nucleus. Scale bars: 100 μm in A to H, and 50 μm in the small inserts.

**Fig 6:**
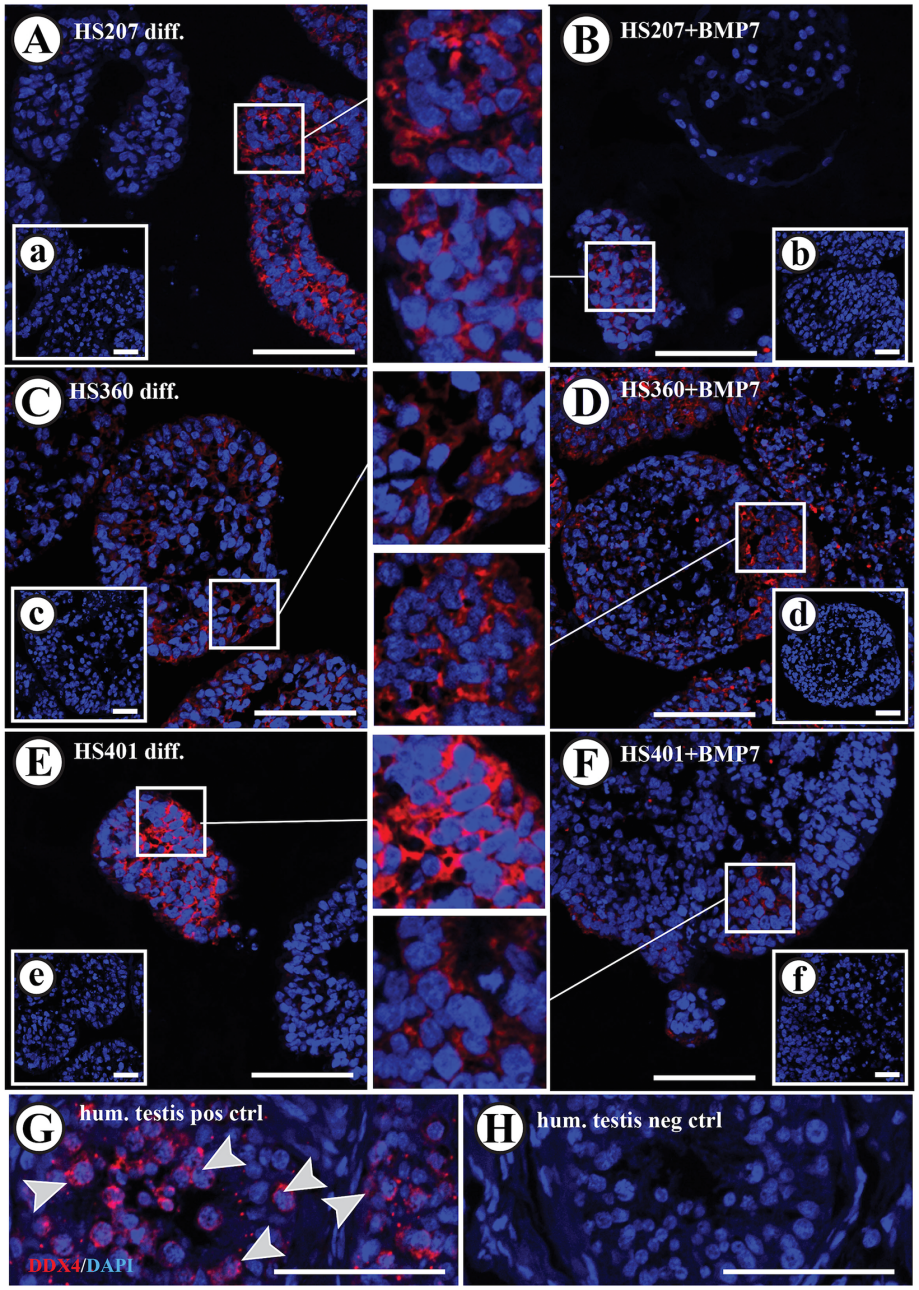
Immunofluorescence detection of DDX4 in HS207, HS360 and HS401 cells cultured in suspension. Cytoplasmic expression of DDX4 was observed in all hES cell lines after spontaneous differentiation (A, C and E) and after stimulation with BMP7 (B, D and F). Cytoplasmic expression of DDX4 was observed in germ cells present in adult human testis (G; arrow heads). Negative controls exhibited no specific staining in either the nucleus or cytoplasm (H and small inserts in A to F). Red: DDX4 staining. Blue: DAPI staining marking the nucleus. Scale bars: 100 μm in A to H, and 50 μm in the small inserts.

Evaluation of SOX9, known as a Sertoli cell marker, protein expression in every cell line following spontaneous differentiation and BMP7 stimulation in suspension cultures (Fig 4) revealed that BMP7 stimulation resulted in the formation of cell colonies surrounded by SOX9-positive cells (Fig 4B, 4D and 4F). This expression profile was observed in 14.9% of all HS207 cell colonies, 11.8% of all HS401 cell colonies and only in 1.5% of all HS360 cell colonies. Only HS401 cells showed this expression pattern after spontaneous differentiation (in 5.0% of all HS401 cell colonies; Fig 4E). Evaluation of WT1 expression was employed to confirm the expression of SOX9 (Fig 5). Nuclear expression of WT1 was observed in cells located at the edge of the spheres following spontaneous differentiation and BMP7 stimulation in suspension culture. Notably, cells that demonstrated a positive expression of SOX9 in the cytoplasm, also revealed expression of WT1 in the nucleus (Fig 5B and 5F). DDX4 protein expression was observed in all cell lines following spontaneous and BMP7-stimulated expression. However, HS207 and HS401 cells showed negative responses to stimulation with BMP7, whereas HS360 cells showed an overall increase in numbers of colonies with cells positive for DDX4 (Table 1 and Fig 6).

**Table 1:** Percentages of hES cell colonies exhibiting cells positive for DDX4.

Cell line/ stimulation	Cell colonies with no DDX4 expression	<25% DDX4-positive cells/colony	25%–75% DDX4-positive cells/colony	75%–100% DDX4-positive cells/colony
**HS207/ None**	5.8%	7.8%	20.4%	66.0%
**HS207/ BMP7**	27.3%	9.1%	30.3%	33.3%
**HS360/ None**	3.7%	6.7%	36.3%	53.3%
**HS306/ BMP7**	2.9%	8.8%	35.3%	52.9%
**HS401/ None**	7.3%	2.4%	20.7%	69.5%
**HS401/ BMP7**	37.8%	8.9%	13.3%	40.0%

Cell colonies were divided into four groups according to their expression profile: no DDX4 expression, <25%, 25%–75% or >75% of DDX4-positive cells present per cell colony. A pool of three experiments (minimum 45 cell colonies) for each cell line and condition was evaluated to define DDX4 expression.

**Fig 7:**
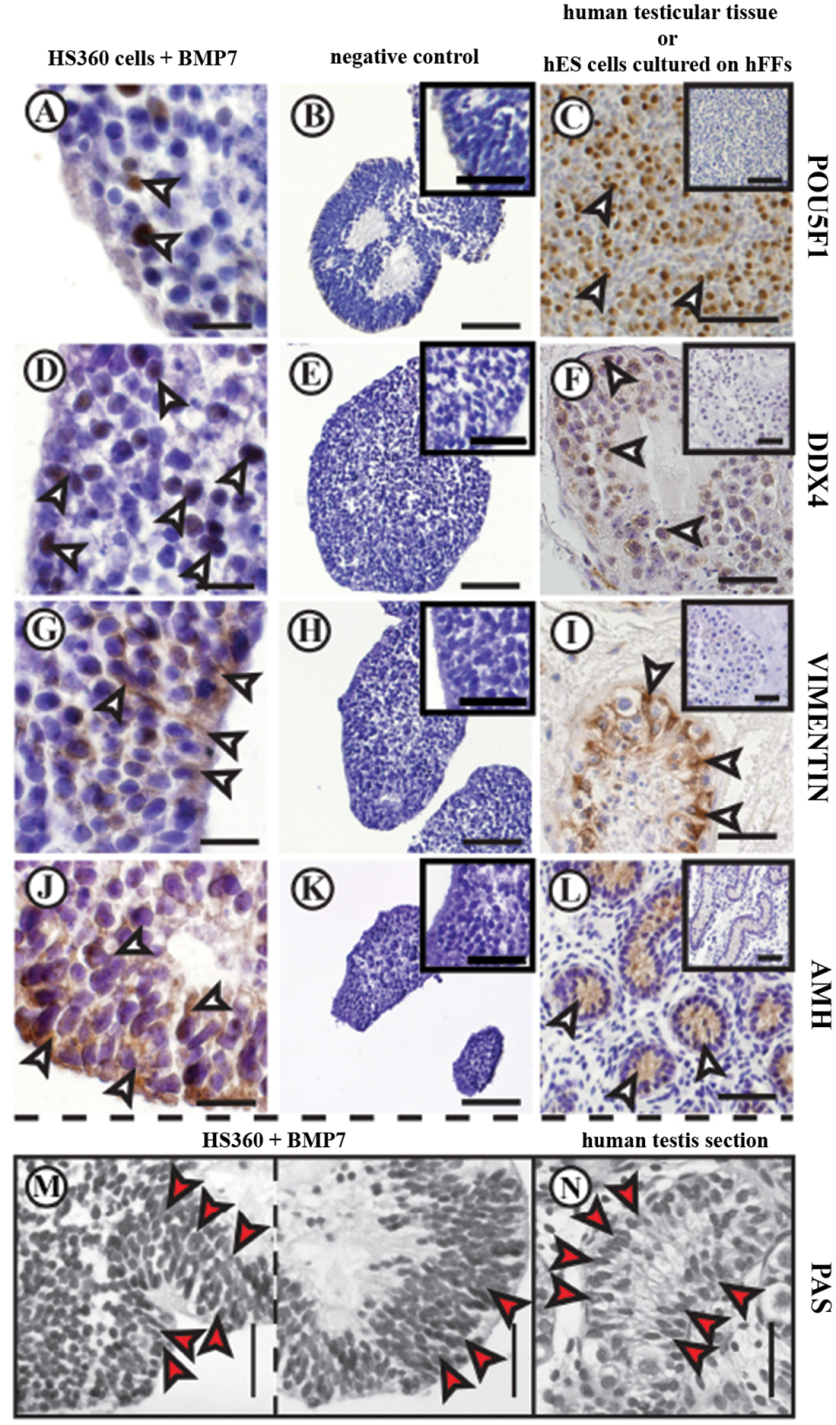
Immunohistochemical detection of POU5F1, DDX4, VIM and AMH in human testis and in HS360 cells cultured with BMP7 in suspension. Nuclear expression of POU5F1 (arrowheads) was observed in BMP7-stimulated HS360 cells after two weeks in culture (A), as well as in undifferentiated hES cells cultured on hFFs (C). Cytoplasmic expression (arrows) of DDX4 (D), VIMENTIN (G) and AMH (J) was present in BMP7-stimulated HS360 cells and human testicular tissue (F, I and L). Negative controls exhibited no specific staining in either the nucleus or cytoplasm (B, E, H and K; and inserts in C, F, I and L). The organization of Sertoli-like cells among stimulated HS360 cells (red arrow heads in M) and Sertoli cells in the one-year-old human testis (red arrow heads in N) was similar. Scale bars: 100 μm in B, C, E, H, K, M and N and the insert in C; 50 μm in F, I and L and the other inserts; 20 μm in A, D, G and J. Abbreviations: AMH: anti-Mullerian hormone, PAS: periodic acid-Schiff. For an explanation of the other abbreviations, see the legend to Fig 2.

Evaluation of protein expression in HS360 cells following BMP7 stimulation in suspension was performed. It was found that POU5F1, DDX4, VIMENTIN, AMH, INSL3 and HS3BD1 were expressed (Fig 7A–7L and S3B Fig). In addition, the organization of Sertoli-like cells in hES cell spheres formed in the presence of BMP7 (Fig 7M) appeared to be similar to the cell organization present in testicular seminiferous cords (Fig 7N). This formation of seminiferous cord-like structures in suspension culture has not been described before.

### Putative Sertoli and Leydig cells do not secrete hormones at this stage of differentiation

To determine whether the putative Sertoli and Leydig cells observed were functional, inhibin B and testosterone levels were assayed in media collected after two days of stimulation with hCG and/or rFSH. The concentration of testosterone in all samples ranged from 2.70±0.46 (SD) to 3.82±0.63 pmol/l, with no significant differences between cell lines or experimental conditions (S5 Fig). Inhibin B was not secreted in detectable amounts under any conditions (limit of detection = 10 pg /ml; S5 Fig). Hence, either these cells do not secrete inhibin B or testosterone in response to stimulation with gonadotropins at this stage of development, or too few functional cells were present.

## Discussion

The major observations documented here are as follows: 1) we confirmed that undifferentiated hES cell lines exhibit different profiles of gene expression. These differences exist between different cell lines and also when a single hES cell line is cultured under different conditions. 2) We observed that both the intended spontaneous and BMP7-stimulated differentiation of the three hES cell lines cultured on hFFs showed no down-regulation of the pluripotency markers *NANOG* and *POU5F1* when compared with the expression profiles of the undifferentiated cells. On the other hand, when differentiated in suspension, these cell lines exhibited the same or down-regulated expression levels of both markers. 3) When HS360 cells were differentiated in suspension, with BMP7 stimulation, we observed that up-regulation of the germ cell markers *KIT* and *DDX4* was accompanied by down-regulation of the pluripotency markers *NANOG* and *POU5F1*. We also observed up-regulation of markers specifically expressed by somatic cells in the human testis (*FSHR* and *HS3BD1*).

### Undifferentiated hES cells demonstrate cell line-specific patterns of gene expression when cultured on hFFs or in suspension

Human ES cells can differentiate towards the germ cell lineage, but with low efficiency [9,16,19,28]. The efficiency of differentiation may be related to the characteristics of individual cell lines or to the culture conditions, or both. Recently, efficient differentiation to early germ cells from hES cells was reported by using non-adherent embryoid body differentiation [39]. However, differentiation was successful only with hES cells maintained in 4i medium, which converts the cells into a distinct pluripotent state.

In addition to conventional hES cell culture on feeder cells, suspension culture conditions can also maintain undifferentiated hES cells [29]. Hence, we wanted to investigate how suspension culture affects hES cells and whether such conditions can “prime” the cells for more efficient germ cell differentiation.

Here, we examined three undifferentiated hES cell lines (all with the XY karyotype) cultured on hFFs and in suspension, both being conditions that can support the undifferentiated growth of these cells. This has been shown previously [28,29] and was confirmed in the present investigation by gene expression analysis. These cell lines expressed the majority of the 90 genes examined in a similar manner, whether cultured on hFFs or in suspension, although the expression of several genes was dependent on the culture conditions. At the same time, the profiles of gene expression in these three undifferentiated hES cell lines differed, mostly with respect to genes related to stemness.

### The NODAL/ACTIVIN pathway as a predictor of hES cell differentiation towards the male germ cell lineage

The NODAL/ACTIVIN signalling pathway has recently been implicated in directing the fate of male gonadal cells in rodents and humans [40–45]. Differences in the expression of related genes might thus be involved in testicular development [44] and in failed germ cell differentiation and cancer formation, e.g., formation of testicular germ cell tumours (TGCTs) [42,43,46], autocrine regulator of proliferation and migration of prostate cancer cells [47], influence on self-renewal and tumorigenicity of pancreatic cancer stem cells [48].

Interestingly we found all crucial genes involved in this pathway (i.e., *EBAF*, *TDGF1*, *LEFTB*, *NODAL* and *GDF3*) to be differently expressed between the hES cell lines when comparing the expression profiles of cells cultured on hFFs and in suspension.

The NODAL/ACTIVIN pathway is thought to help to maintain early male primordial germ cells (PGCs) in an undifferentiated state, to ensure that they do not undergo meiosis, but rather differentiate towards male gonocytes [42]. In addition, early Sertoli cell differentiation and gonadal cord formation also appear to involve the NODAL/ACTIVIN pathway [41,44]. *EBAF* and *LEFTB* inhibit *NODAL*, whereas *TDGF1* activates this pathway [42,43]. *GDF3* potentiates *NODAL* activity, but has also been related to the development of testicular cancer formation [46,49].

In HS401 cells cultured in suspension, the expression of all genes whose products are involved in the NODAL/ACTIVIN pathway was down-regulated, whereas in HS207 cells under these same conditions, expression of *TDGF1* was up-regulated, while that of *LEFTB*, *NODAL* and *EBAF* was down-regulated. In contrast, HS360 cells in suspension exhibited attenuated expression of *LEFTB* and *GDF3*. The products of these genes inhibit and potentiate the expression of *NODAL*, respectively. Thus, HS360 appeared to be the only cell line among the three examined that did not show any pronounced alteration in the expression of genes related to the NODAL/ACTIVIN pathway. Therefore it was identified as being the most promising candidate among the tested hES cell lines for further differentiation into gonadal cells. In line with this suggestion, other genes of importance as regards the early development of germ cells were up-regulated in HS360 cells. These are *LIN28* and *KIT*, which are both crucial for PGC differentiation [50], and *SOX2*, which is required for PGC proliferation and stimulation of *KIT*, but which is not expressed by PGCs [51–53].

### BMP7 evokes down-regulation of the expression of mRNAs encoding pluripotency markers in cultures of hES cells in suspension, but not on hFFs

To test whether differences in gene expression by undifferentiated hES cells can predict their potential for differentiation towards the male germ lineage, we allowed these cells to differentiate both spontaneously (in the absence of exogenous rFGF2) as well as in response to stimulation by BMP7. Upon differentiation in suspension, the expression of mRNAs encoding pluripotency markers by the hES cells was dramatically decreased. In contrast, with attempted differentiation on hFFs, this expression remained as high or was even higher than that in undifferentiated hES cells. This could be explained by secretion of factors such as FGF2 and IL6 (interleukin 6) by the hFFs, which could influence the ability of the hES cells to remain pluripotent. Recent studies have revealed that the secretion of FGF2 by hFFs is sufficient for the culture of hES cells without exogenous FGF2 [37,38].

### BMP7 stimulation results in up-regulation of the expression of mRNAs encoding germ and somatic testicular cell markers in cultures of hES cells both in suspension and on hFFs

We observed low expression of *DDX4* mRNA in undifferentiated hES cells, as also shown by others [7,9,18,27,28]. This expression was up-regulated significantly in HS360 and HS401 cells after 14 days of BMP7-stimulated differentiation, both on hFFs and in suspension. The significantly increased expression of *DDX4* after stimulation was accompanied by elevated expression of *KIT* in suspension cultures of HS360 cells only, whereas BMP7 stimulation resulted in higher expression profiles of *KIT* when compared with spontaneous differentiation in all tested cell lines cultured on hFFs. Following differentiation in suspension, DDX4 protein was detected primarily in cells situated at the edges of the spheres and no DDX4 protein was observed in undifferentiated hES cells.

In addition to the above, expression of FSHR was up-regulated in HS207 and HS360 cell lines upon stimulation with BMP7. With respect to genes expressed by somatic cells of the male gonad, upon BMP7-stimulated and spontaneous differentiation in suspension, we observed general up-regulation of markers specific for Sertoli and Leydig cells at the mRNA level. Along with the establishment of PGCs, Sertoli cells play an important role at the time of sex determination [54,55]. WT1 and SOX9 are known to be expressed in immature Sertoli cells and are important factors for Sertoli cell differentiation and subsequent testicular cord formation [56]. SOX9 is expressed in the cytoplasm of pre-Sertoli cells as long as they are in the bi-potential state [56,57]. Around week 6.5 in humans, the expression of SOX9 is translocated to the nucleus of Sertoli cells in males, but its expression decreases over time in females [56].

Secretion of AMH, a specific marker of immature Sertoli cells, is reduced drastically during puberty [58], and this protein was expressed strongly by both stimulated and un-stimulated cells. VIM filaments play a major role in the cytoskeletal structure of Sertoli cells [59], probably by interacting with germ cells in the seminiferous tubules [60]. However, since VIM is not a specific marker for Sertoli cells, the Sertoli cell identity needs to be confirmed by other markers such as FSHr, SOX9, AMH or WT1. The putative Sertoli cells immunostained positively for SOX9, VIM, WT1 and AMH proteins and were localized primarily at the edges of the spheres i.e., the same region harbouring cells positive for DDX4 protein. The putative Leydig cells immunostained positively for both INSL3 [61,62] and HSD3B1 [61–63] proteins.

### hES cells that differentiate in suspension in response to exogenous BMP7 exhibit morphology resembling that of seminiferous cords in normal male gonads and they also express testicular-specific markers

hES cells that spontaneously differentiated in suspension cultures showed morphology similar to that of immature somatic cells (e.g., immature Sertoli cells: irregular elongating nuclei, one or two prominent nucleoli, dark karyoplasm) usually found in seminiferous cords of the human testis. The ultrastructures of these putative germ and Sertoli cells and possible connections via cytoplasmic bridges require further investigation.

Tests of functionality of these putative gonadal somatic cells revealed low levels of inhibin B and testosterone expression (at or below the limit of detection of the assays used) in the culture media following stimulation with rFSH and hCG. This could mean either that the cells were not yet fully functional or that only a few cells were functional. In either case, protocols need to be optimized further with the goal of obtaining functional cells.

## Conclusion

In conclusion, we show here for the first time that a combination of different culture conditions together with gene analysis can be used to predict the cell line-specific potential of human embryonic stem cells to differentiate towards the male germ cell lineage and somatic gonadal cell types. Expression patterns of mRNA, including up-regulation of early germ cell markers, combined with down-regulation of markers expressed in the mesoderm, endoderm and ectoderm, as well as pluripotency markers, seem to be the most promising signs in hES cell lines of further differentiation towards the male germ lineage. In addition, our analysis revealed that the cell line exhibiting this profile showed only minor changes in the NODAL/ACTIVIN signalling pathway, which might serve as a gatekeeper as regards male germ cell differentiation even *in vitro*. Furthermore, we found that hES cells showing the above expression profile exhibit levels of mRNA and proteins, and morphology, similar to those of early male gonadal cells found *in vivo*, when stimulated with BMP7 and cultured in suspension. Maintaining hES cells in a pluripotent state using suspension culture conditions might keep them better primed for germ cell differentiation. This approach may be of value in obtaining mature functional male germ cells from hES cells and in providing new insight into the molecular and cellular mechanisms underlying human spermatogenesis.

## Supporting Information

S1 Fig Morphology of HS360 cells cultured on hFFs and in suspension. Undifferentiated HS360 cells grown on mitotically inactivated hFFs (asterisk) exhibit normal hES cell morphology including formation of flat and compact colonies with sharp edges (arrowhead) (A; 40x magnification). In suspension culture, these cells form compact spheres (B and C; 40x magnification and 200x magnification, respectively). hFFs: human foreskin fibroblasts.
(TIF)

S2 Fig RT-PCR analysis revealed that the three undifferentiated hES cell lines (HS207, HS360 and HS401) express a panel of consensus markers (*NANOG*, POU5F1, GDF3, GABRB3 and TDGF1) for pluripotency when cultured either on hFFs or as spheres in suspension. *ACTB*: endogenous control. hFFs: human foreskin fibroblasts. A list of gene names and abbreviations can be found in S6 Table.
(TIF)

S3 Fig Expression of the Leydig cells markers INSL3 and HSD3B1 by HS360 cells cultured in suspension. (A) Expression at the mRNA level. hESCs: undifferentiated human embryonic stem cells; diff hESCs: spontaneously differentiated hESCs; BMP7 hESCs: hESCs stimulated to differentiate by BMP7. The relative level of mRNA was calculated by the ddCt procedure from the mean of triplicates and statistical analysis was performed by way of One-way RM ANOVA. *p <0.05 in comparison with both the undifferentiated and spontaneously differentiated cells. (B) Immunohistochemical staining revealed cytoplasmic expression (arrows) of both INSL3 and HSD3B1 in HS360 cells stimulated by BMP7 (I and II) as well as in human testicular tissue (III and IV). Negative controls (inserts) exhibited no specific staining. Scale bars: 50 μm. hESCs: human embryonic stem cells; BMP7: bone morphogenetic protein 7. A list of gene names and abbreviations can be found in S6 Table.
(TIF)

S4 Fig Transmission electron micrographs of undifferentiated HS401 cells and HS401 cells differentiated in response to BMP7. (A) Undifferentiated cells exhibit characteristic morphology, with highly condensed nucleoli (N) and elongated mitochondria (M). (B) BMP7-stimulated cells exhibit a morphology similar to that of hES cells during early differentiation with charateristic highly condensed nucleoli (N), round mitochondria (M) and lipid droplets (L). hES cells: human embryonic stem cells; BMP7: bone morphogenetic protein 7.
(TIF)

S5 Fig Levels of testosterone and inhibin B in the media of hES cells cultured in suspension. HS207, HS360 and HS401 cells that differentiated in suspension spontaneously (spon) or in repsonse to BMP7 (B7) were then stimulated for two days with rFSH and/or hCG. (A) Testosterone levels, (B) Levels of inhibin B. The limits of detection of the testosterone (0.137 nmol/l) and inhibin B (10 pg/ml) assays are depicted as grey lines. hES cells: human embryonic stem cells; BMP7: bone morphogenetic protein 7; rFSH: recombinant follicle-stimulating hormone; hCG: chorionic gonadotrophin.
(TIF)

S1 Table Sequences of the primers used for RT-PCR (previously described by Awan *et al*., 2010). All primer pairs exhibited a melting temperature of 60–62°C. fw: forward primer; rev: reverse primer; bp: base pairs. A list of gene names and abbreviations can be found in S6 Table.
(DOC)

S2 Table TaqMan probes employed in Q-PCR analysis. All of these had a melting temperature close to 60°C. A list of gene names and abbreviations can be found in S6 Table.
(DOC)

S3 Table Sequences of primers employed for SYBR Green Q-PCR. All primer pairs exhibited a melting temperature close to 60°C. fw: forward primer; rev: reverse primer. A list of gene names and abbreviations can be found in S6 Table.
(DOC)

S4 Table Specific primary antibodies and control IgGs used for immunohistochemical staining. IgG: immunoglobulin G; DDX4: DEAD (Asp-Glu-Ala-Asp) box polypeptide 4; POU5F1: POU class 5 homeobox 1; AMH: anti-Mullerian hormone; VIM: vimentin; HSD3B1: hydroxy-delta-5-steroid dehydrogenase; INSL3: insulin-like DNA sequence; anti-SOX9: SRY (sex determining region Y)-box 9. WT1: Wilms tumor 1.
(DOC)

S5 Table Biotin-labelled secondary antibodies utilized for immunohistochemical staining. IgG: immunoglobulin G.
(DOC)

S6 Table Abbreviations and names of all genes included in the TLDA evaluation (Fig 1 and S7 and S8 Tables), qPCR analysis (Figs 2 and 3, S3 Fig and S2 and S3 Tables) and RT-PCR analysis (S2 Fig and S1 Table). (DOCX)

S7 Table Comparison of gene expression by hES cells cultured in suspension versus hES cells cultured on human foreskin fibroblasts. A list of gene names and abbreviations can be found in S6 Table.
(DOC)

S8 Table Summary of results of statistical testing between cell lines as regards to TaqMan Low-Density Array data. A list of gene names and abbreviations can be found in S6 Table.
(DOC)
