# Supplementary figures and images for: A Combination of Culture Conditions and Gene Expression Analysis Can Be Used to Investigate and Predict hES Cell Differentiation Potential towards Male Gonadal Cells
Source: PLoS One. 2015 Dec 2;10(12):e0144029. doi: 10.1371/journal.pone.0144029 (PMC4667967; doi:10.1371/journal.pone.0144029)

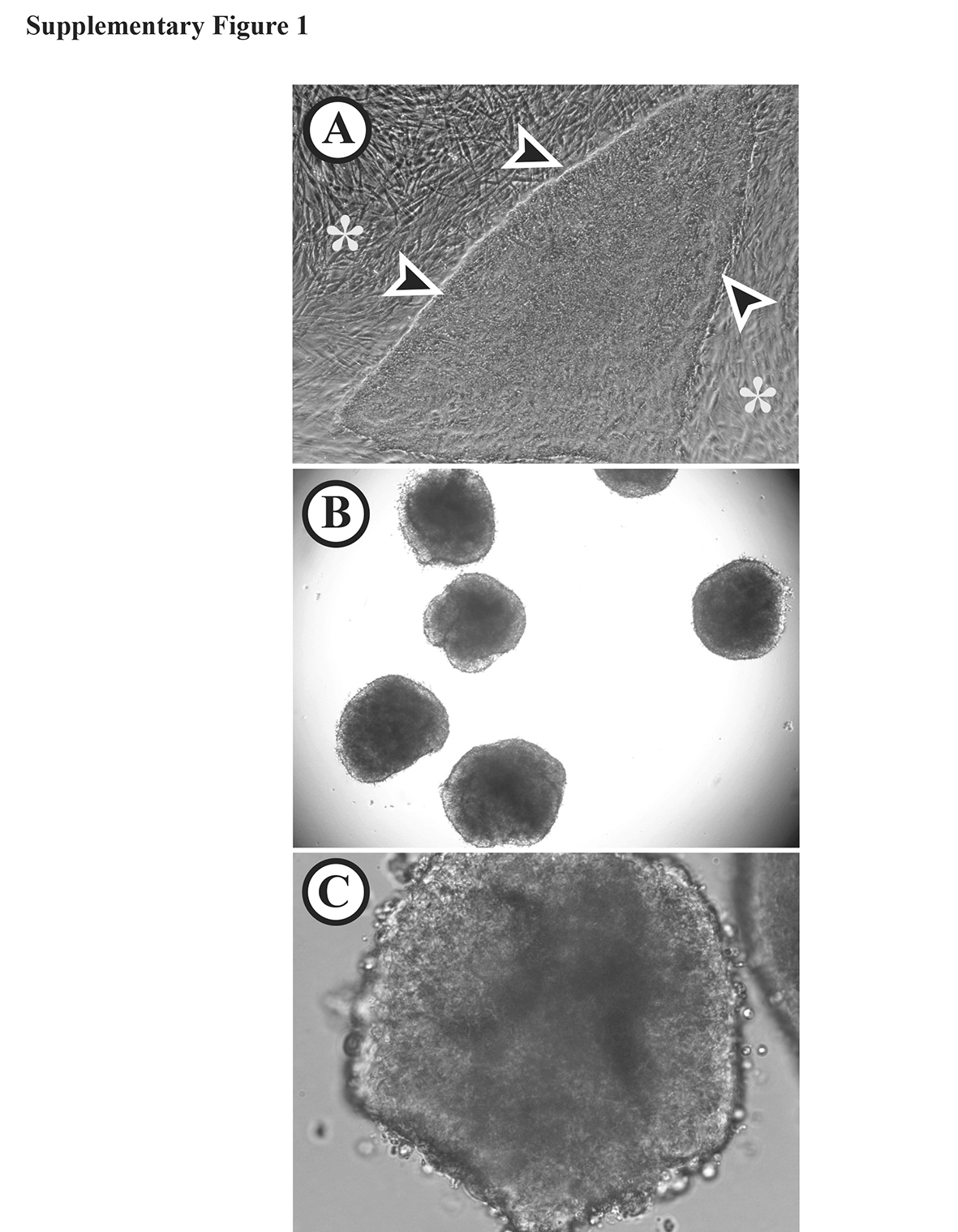

Supplement: S1 Fig — Undifferentiated HS360 cells grown on mitotically inactivated hFFs (asterisk) exhibit normal hES cell morphology including formation of flat and compact colonies with sharp edges (arrowhead) (A; 40x magnification). In suspension culture, these cells form compact spheres (B and C; 40x magnification and 200x magnification, respectively). hFFs: human foreskin fibroblasts. (TIF) [file pone.0144029.s001.tif]

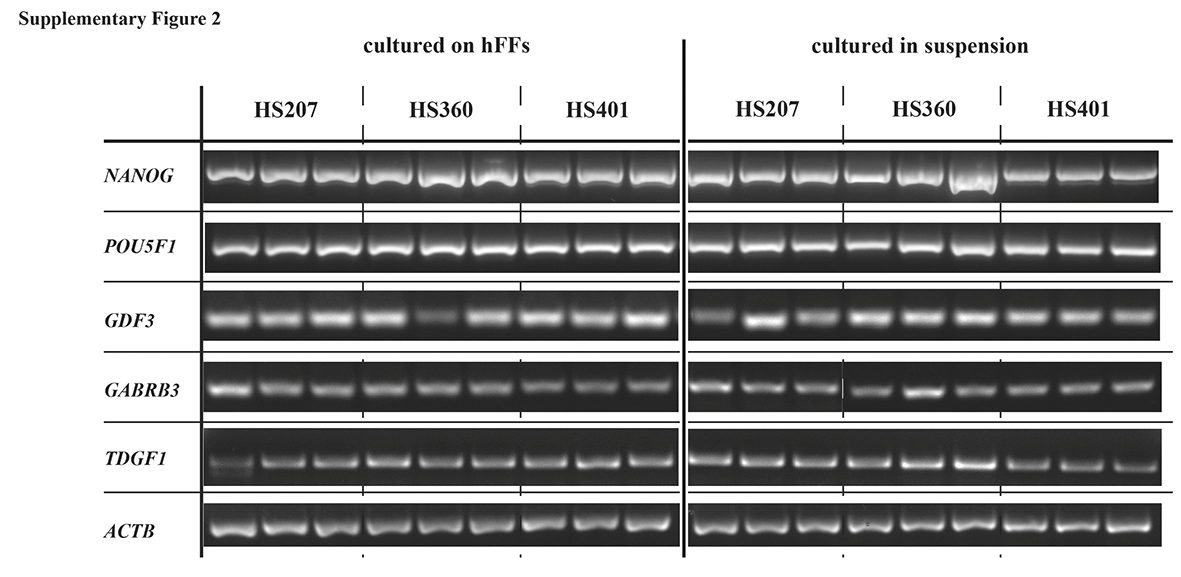

Supplement: S2 Fig — ACTB: endogenous control. hFFs: human foreskin fibroblasts. A list of gene names and abbreviations can be found in S6 Table. (TIF) [file pone.0144029.s002.tif]

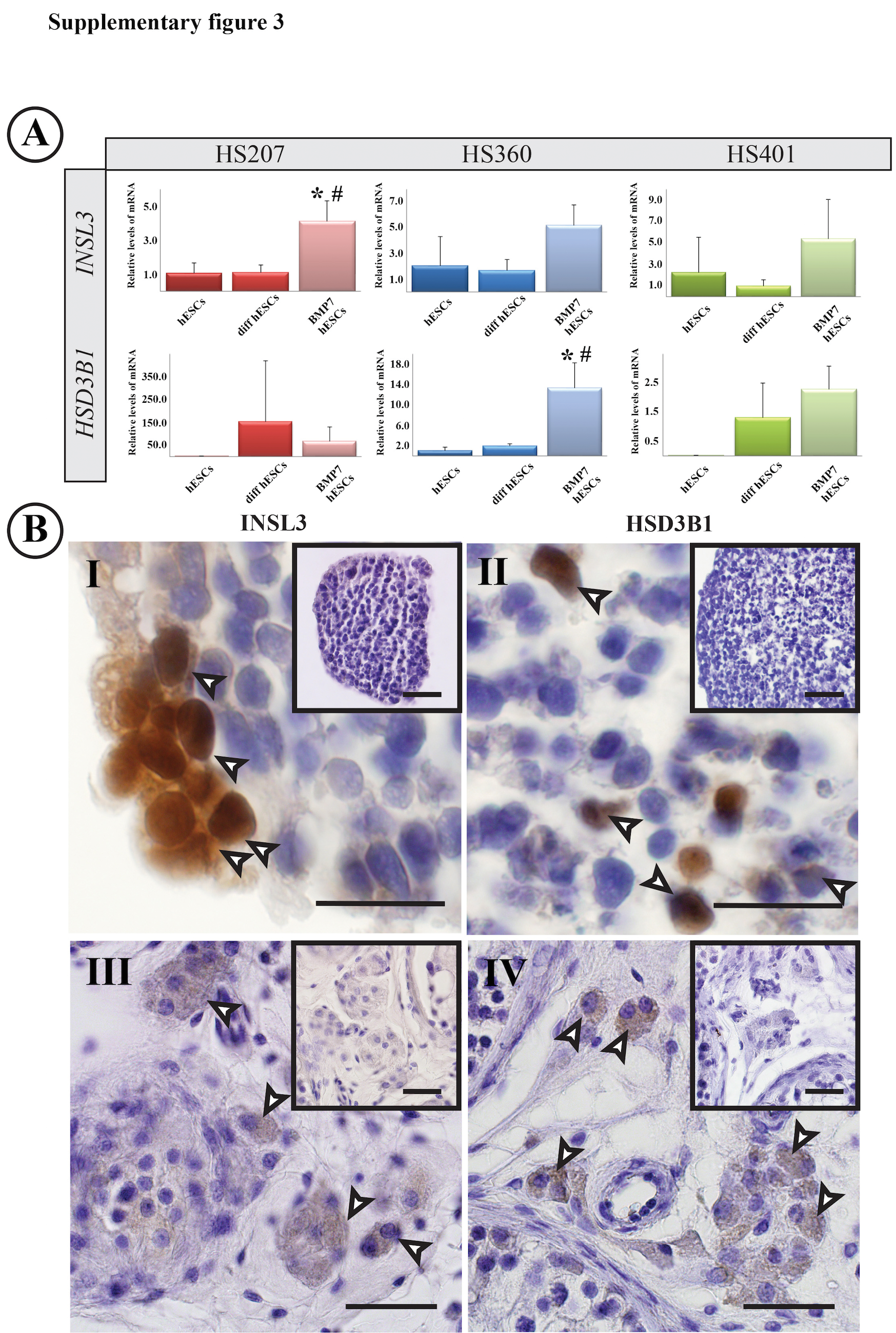

Supplement: S3 Fig — (A) Expression at the mRNA level. hESCs: undifferentiated human embryonic stem cells; diff hESCs: spontaneously differentiated hESCs; BMP7 hESCs: hESCs stimulated to differentiate by BMP7. The relative level of mRNA was calculated by the ddCt procedure from the mean of triplicates and statistical analysis was performed by way of One-way RM ANOVA. *p <0.05 in comparison with both the undifferentiated and spontaneously differentiated cells. (B) Immunohistochemical staining revealed cytoplasmic expression (arrows) of both INSL3 and HSD3B1 in HS360 cells stimulated by BMP7 (I and II) as well as in human testicular tissue (III and IV). Negative controls (inserts) exhibited no specific staining. Scale bars: 50 μm. hESCs: human embryonic stem cells; BMP7: bone morphogenetic protein 7. A list of gene names and abbreviations can be found in S6 Table. (TIF) [file pone.0144029.s003.tif]

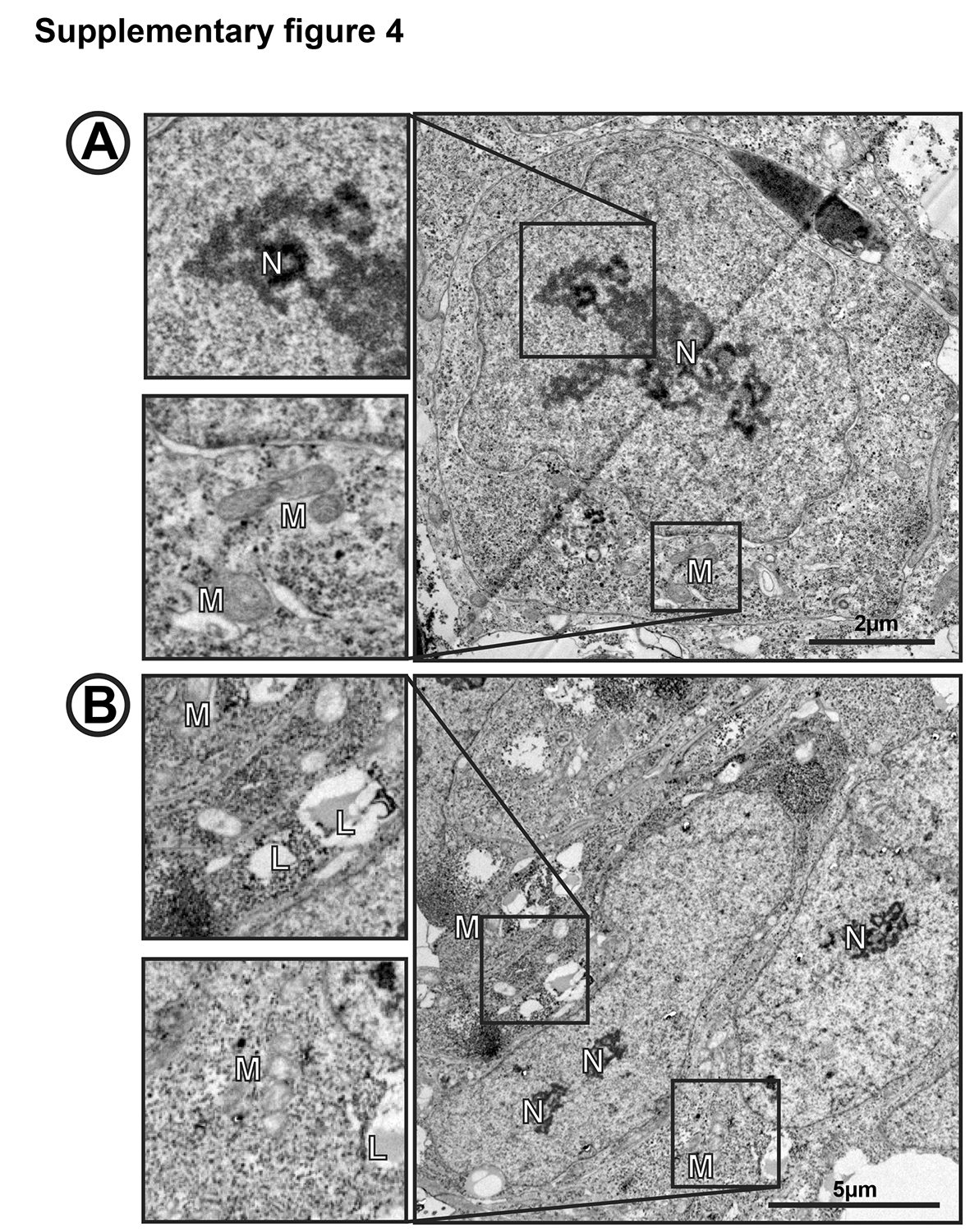

Supplement: S4 Fig — (A) Undifferentiated cells exhibit characteristic morphology, with highly condensed nucleoli (N) and elongated mitochondria (M). (B) BMP7-stimulated cells exhibit a morphology similar to that of hES cells during early differentiation with charateristic highly condensed nucleoli (N), round mitochondria (M) and lipid droplets (L). hES cells: human embryonic stem cells; BMP7: bone morphogenetic protein 7. (TIF) [file pone.0144029.s004.tif]

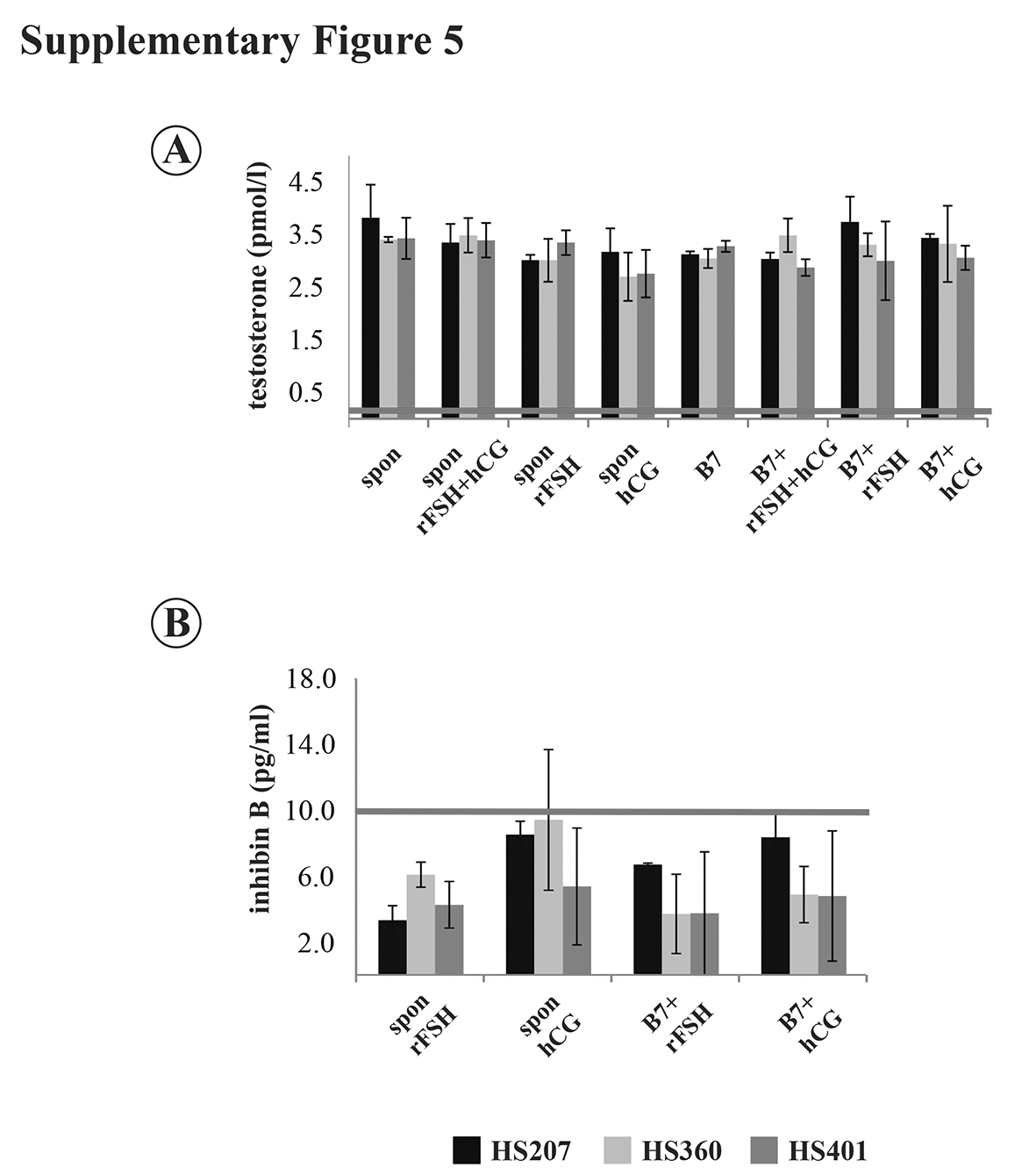

Supplement: S5 Fig — HS207, HS360 and HS401 cells that differentiated in suspension spontaneously (spon) or in repsonse to BMP7 (B7) were then stimulated for two days with rFSH and/or hCG. (A) Testosterone levels, (B) Levels of inhibin B. The limits of detection of the testosterone (0.137 nmol/l) and inhibin B (10 pg/ml) assays are depicted as grey lines. hES cells: human embryonic stem cells; BMP7: bone morphogenetic protein 7; rFSH: recombinant follicle-stimulating hormone; hCG: chorionic gonadotrophin. (TIF) [file pone.0144029.s005.tif]
