# Supplementary material for: A Combination of Culture Conditions and Gene Expression Analysis Can Be Used to Investigate and Predict hES Cell Differentiation Potential towards Male Gonadal Cells
Source: PLoS One. 2015 Dec 2;10(12):e0144029. doi: 10.1371/journal.pone.0144029 (PMC4667967; doi:10.1371/journal.pone.0144029)
Supplement: S1 Table — All primer pairs exhibited a melting temperature of 60–62°C. fw: forward primer; rev: reverse primer; bp: base pairs. A list of gene names and abbreviations can be found in S6 Table. (DOC) [file pone.0144029.s006.doc]

Supplementary Table 1:

| **mRNA species** | **Primer sequence (5´→3´)** | **Amplicon size (bp)** |
| --- | --- | --- |
| ***NANOG*** | Fw: CAAAGGCAAACAACCCACTT  Rev: CTGGATGTTCTGGGTCTGGT | 426 |
| ***POU5F1*** | Fw: GACAACAATGAAAATCTTCAGGAGA Rev: TTCTGGCGCCGGTTACAGAACCA | 218 |
| ***TDGF1*** | Fw: AGCACAGTAAGGAGCTAAACA  Rev: CAGTTCCGTCCGTAGAAGGAG | 101 |
| ***GDF3*** | Fw: GTACTTCGCTTTCTCCCAGAC  Rev: GCCAATGTCAACTGTTCCCTT | 131 |
| ***GABRB3*** | Fw: CAAGCTGTTGAAAGGCTACGA  Rev: ACTTCGGAAACCATGTCGATG | 108 |
| ***ACTB*** | Fw: CCTGGCACCCAGCACAAT  Rev: GGGCCGGACTCGTCATAC | 144 |
