# Supplementary material for: A Combination of Culture Conditions and Gene Expression Analysis Can Be Used to Investigate and Predict hES Cell Differentiation Potential towards Male Gonadal Cells
Source: PLoS One. 2015 Dec 2;10(12):e0144029. doi: 10.1371/journal.pone.0144029 (PMC4667967; doi:10.1371/journal.pone.0144029)
Supplement: S2 Table — All of these had a melting temperature close to 60°C. A list of gene names and abbreviations can be found in S6 Table. (DOC) [file pone.0144029.s007.doc]

Supplementary Table 2:

| **mRNA species** | **Assay ID** |
| --- | --- |
| ***NANOG*** | Hs02387400_g1 |
| ***DDX4*** | Hs00251833_m1 |
| ***FSHR*** | Hs00174865_m1 |
| ***VIM*** | Hs00185584_m1 |
| ***HSD3B1*** | Hs04194787_g1 |
| ***INSL3*** | Hs01394273_m1 |
| ***GAPDH*** | Hs02758991_g1 |
