# Supplementary material for: A Combination of Culture Conditions and Gene Expression Analysis Can Be Used to Investigate and Predict hES Cell Differentiation Potential towards Male Gonadal Cells
Source: PLoS One. 2015 Dec 2;10(12):e0144029. doi: 10.1371/journal.pone.0144029 (PMC4667967; doi:10.1371/journal.pone.0144029)
Supplement: S3 Table — All primer pairs exhibited a melting temperature close to 60°C. fw: forward primer; rev: reverse primer. A list of gene names and abbreviations can be found in S6 Table. (DOC) [file pone.0144029.s008.doc]

Supplementary Table 3:

| **mRNA species** | **Primer sequence (5´→3´)** |
| --- | --- |
| ***POU5F1*** | Fw: GACAACAATGAAAATCTTCAGGAGA  Rev: TTCTGGCGCCGGTTACAGAACCA |
| ***KIT*** | Fw: GGCATGCTCCAATGTGTGG  Rev: GGTGTGGGGATGGATTTGC |
| ***GAPDH*** | Fw: GAAGGTGAAGGTCGGAGTCAAC  Rev: CAGAGTTAAAAGCAGCCCTGG |
