# Supplementary material for: A Combination of Culture Conditions and Gene Expression Analysis Can Be Used to Investigate and Predict hES Cell Differentiation Potential towards Male Gonadal Cells
Source: PLoS One. 2015 Dec 2;10(12):e0144029. doi: 10.1371/journal.pone.0144029 (PMC4667967; doi:10.1371/journal.pone.0144029)
Supplement: S4 Table — IgG: immunoglobulin G; DDX4: DEAD (Asp-Glu-Ala-Asp) box polypeptide 4; POU5F1: POU class 5 homeobox 1; AMH: anti-Mullerian hormone; VIM: vimentin; HSD3B1: hydroxy-delta-5-steroid dehydrogenase; INSL3: insulin-like DNA sequence; anti-SOX9: SRY (sex determining region Y)-box 9. WT1: Wilms tumor 1. (DOC) [file pone.0144029.s009.doc]

| **Antibodies** | **Raised in, type** | **Concentration (mg/ml)** | **Obtained from (catalogue number, company)** | **Blocking serum employed** |
| --- | --- | --- | --- | --- |
| **Anti-DDX4** | rabbit, polyclonal | - 1. (IHC)   0.002 (IF) | ab13840, AbCam | goat (IHC)  chicken (IF) |
| **Anti-POU5F1** | goat, polyclonal | 0.004 | SC-8629, Santa Cruz | horse |
| **Anti-AMH** | rabbit, polyclonal | 0.005 | ab103233, AbCam | goat |
| **Anti-VIM** | rabbit, polyclonal | 0.05 | ab8545, AbCam | goat |
| **Anti-HSD3B1** | mouse, monoclonal | 0.002 | SC-100466, Santa Cruz | horse |
| **Anti-INSL3** | rabbit, polyclonal | 0.004 | SC-134586, Santa Cruz | goat |
| **Anti-SOX9** | rabbit, polyclonal | 0.002 | AB5535, Millipore | chicken |
| **Anti-WT1** | rabbit, polyclonal | 0.002 | ab89901, AbCam | donkey |
| **IgG** | goat | same as for the specific primary antibody | SC-2028, Santa Cruz | horse |
| **IgG** | mouse | same as for the specific primary antibody | SC-2025, Santa Cruz | horse |
| **IgG** | rabbit | same as for the specific primary antibody | SC-2027, Santa Cruz | goat |
| **IgG** | rabbit | same as for the specific primary antibody (IF) | ab27478, AbCam | chicken |
