# Supplementary material for: A Combination of Culture Conditions and Gene Expression Analysis Can Be Used to Investigate and Predict hES Cell Differentiation Potential towards Male Gonadal Cells
Source: PLoS One. 2015 Dec 2;10(12):e0144029. doi: 10.1371/journal.pone.0144029 (PMC4667967; doi:10.1371/journal.pone.0144029)
Supplement: S5 Table — IgG: immunoglobulin G. (DOC) [file pone.0144029.s010.doc]

| **Raised in** | **Raised against** | **Concentration (mg/ml)** | **Obtained from (catalogue number, company)** |
| --- | --- | --- | --- |
| **goat** | rabbit IgG | 0.005 | BA1000, Vector Lab |
| **horse** | goat IgG | 0.005 | BA9500, Vector Lab |
| **horse** | mouse IgG | 0.005 | BA-200, Vector Lab |
| **chicken** | rabbit IgG | 0.002 | Sc-2963, Santa Cruz |
| **donkey** | rabbit IgG | 0.005 | 711-166-152, Jackson Immunoresearch |
