# Supplementary material for: A Combination of Culture Conditions and Gene Expression Analysis Can Be Used to Investigate and Predict hES Cell Differentiation Potential towards Male Gonadal Cells
Source: PLoS One. 2015 Dec 2;10(12):e0144029. doi: 10.1371/journal.pone.0144029 (PMC4667967; doi:10.1371/journal.pone.0144029)
Supplement: S6 Table — (DOCX) [file pone.0144029.s011.docx]

| **Gene symbol** | **Name** |
| --- | --- |
| *ACTB* | *actin beta* |
| *ACTC* | *actin, alpha, cardiac muscle 1* |
| *AFP* | *alpha-fetoprotein* |
| *BRIX* | *BRX1, biogenesis of ribosomes, homolog* |
| *CD34* | *CD34 molecule* |
| *CD9* | *CD9 molecule* |
| *CDH5* | *cadherin 5, type 2 (vascular endothelium)* |
| *CDX2* | *caudal type homeobox 2* |
| *CGB* | *chorionic gonadotropin, beta polypeptide* |
| *COL1A1* | *collagen, type I, alpha 1* |
| *COL2A1* | *collagen, type II, alpha 1* |
| *COMMD3* | *COMM domain containing 3* |
| *CRABP2* | *cellular retinoic acid binding protein 2* |
| *DDX4* | *DEAD (Asp-Glu-Ala-Asp) box polypeptide 4* |
| *DES* | *desmin* |
| *DNMT3B* | *DNA (cytosine-5-)-methyltransferase 3 beta* |
| *EBAF (*also known as *LEFTY1)* | *left-right determination factor 1* |
| *EOMES* | *eomesodermin* |
| *FGF4* | *fibroblast growth factor 4* |
| *FGF5* | *fibroblast growth factor 5* |
| *FLT1* | *fms-related tyrosine kinase 1* |
| *FN1* | *fibronectin 1* |
| *FOXA2* | *forkhead box A2* |
| *FOXD3* | *forkhead box D3* |
| *FSHR* | *Follicle-stimulating hormone receptor* |
| *GABRB3* | *gamma-aminobutyric acid (GABA) A receptor, beta 3* |
| *GAL* | *galanin/GMAP prepropeptide* |
| *GAPDH* | *glyceraldehyde-3-phosphate dehydrogenase* |
| *GATA4* | *GATA binding protein 4* |
| *GATA6* | *GATA binding protein 6* |
| *GBX2* | *gastrulation brain homeobox 2* |
| *GDF3* | *growth differentiation factor 3* |
| *GFAP* | *glial fibrillary acidic protein* |
| *GRB7* | *growth factor receptor-bound protein 7* |
| *HBZ* | *hemoglobin, zeta* |
| *HLXB9 (*also known as *MNX1)* | *motor neuron and pancreas homeobox 1* |
| *HSD3B1* | hydroxy-delta-5-steroid dehydrogenase |
| *IAPP* | *islet amyloid polypeptide* |
| *IFITM1* | *interferon induced transmembrane protein 1* |
| *IFITM2* | *interferon-induced transmembrane protein 2* |
| *IL6ST* | *interleukin 6 signal transducer (gp130, oncostatin M receptor)* |
| *IMP2 (*also known as *IGF2BP2)* | *insulin-like growth factor 2 mRNA binding protein 2* |
| *INSL* | insulin-like 3 |
| *ISL1* | *ISL LIM homeobox 1* |
| *KIT* | *v-kit Hardy-Zuckerman 4 feline sarcoma viral oncogene homolog* |
| *LAMA1* | *laminin, alpha 1* |
| *LAMB1* | *laminin, beta 1* |
| *LAMC1* | *laminin, gamma 1* |
| *LEFTB (*also known as *LEFTY2)* | *left-right determination factor 2* |
| *LIFR* | *leukemia inhibitory factor receptor alpha* |
| *LIN28* | *lin-28 homolog A* |
| *MYF5* | *myogenic factor 5* |
| *MYOD1* | *myogenic differentiation 1* |
| *NANOG* | *Nanog homeobox* |
| *NES* | *nestin* |
| *NEUROD1* | *neuronal differentiation 1* |
| *NODAL* | *nodal growth differentiation factor* |
| *NOG* | *noggin* |
| *NR5A2* | *nuclear receptor subfamily 5, group A, member 2* |
| *NR6A1* | *nuclear receptor subfamily 6, group A, member 1* |
| *OLIG2* | *oligodendrocyte lineage transcription factor 2* |
| *PAX4* | *paired box 4* |
| *PAX6* | *paired box 6* |
| *PECAM1* | *platelet/endothelial cell adhesion molecule 1* |
| *PODXL* | *podocalyxin-like* |
| *POU5F1* | *POU class 5 homeobox 1* |
| *PTEN* | *phosphatase and tensin homolog* |
| *RAF1* | *v-raf-1 murine leukaemia viral oncogene homolog 1* |
| *REST* | *RE1-silencing transcription factor* |
| *RUNX2* | *runt-related transcription factor 2* |
| *SEMA3A* | *sema domain, immunoglobulin domain (Ig), short basic domain, secreted, (semaphorin) 3A* |
| *SERPINA1* | *serpin peptidase inhibitor, clade A (alpha-1 antiproteinase, antitrypsin), member 1* |
| *SFRP2* | *secreted frizzled-related protein 1* |
| *SOX17* | *SRY (sex determining region Y)-box 17* |
| *SOX2* | *SRY (sex determining region Y)-box 2* |
| *SST* | *somatostatin* |
| *SYCP3* | *synaptonemal complex protein 3* |
| *SYP* | *synaptophysin* |
| *T* | *T, brachyury homolog (mouse)* |
| *TAT* | *tyrosine aminotransferase* |
| *TDGF1* | *teratocarcinoma-derived growth factor 1* |
| *TERT* | *telomerase reverse transcriptase* |
| *TFCP2L1* | *transcription factor CP2-like 1* |
| *UTF1* | *undifferentiated embryonic cell transcription factor 1* |
| *VIM* | *Vimentin* |
| *WT1* | *Wilms tumour 1* |
| *XIST* | *X inactive specific transcript (non-protein coding)* |
| *ZFP42* | *ZFP42 zinc finger protein* |
