# Supplementary material for: A Combination of Culture Conditions and Gene Expression Analysis Can Be Used to Investigate and Predict hES Cell Differentiation Potential towards Male Gonadal Cells
Source: PLoS One. 2015 Dec 2;10(12):e0144029. doi: 10.1371/journal.pone.0144029 (PMC4667967; doi:10.1371/journal.pone.0144029)
Supplement: S7 Table — A list of gene names and abbreviations can be found in S6 Table. (DOC) [file pone.0144029.s012.doc]

| **GENE** | **GROUP** | p value | p value summary | Significantly different? (p < 0.05) | Up/Down-regulation |
| --- | --- | --- | --- | --- | --- |
| ***ACTC*** | Mesoderm | 0.0258 | * | Yes | down |
| ***AFP*** | Endoderm | 0.0178 | * | Yes | up |
| ***BRIX*** | Stem Cell | < 0.0001 | **** | Yes | up |
| ***CD34*** | Endoderm | 0.0602 | ns | No | N/A |
| ***CD9*** | Stem Cell | 0.0547 | ns | No | N/A |
| ***CDH5*** | Endoderm | 0.3372 | ns | No | N/A |
| ***CDX2*** | Trophoblast | 0.0287 | * | Yes | up |
| ***CGB*** | Trophoblast | 0.6752 | ns | No | N/A |
| ***COL1A1*** | Mesoderm | < 0.0001 | **** | Yes | down |
| ***COL2A1*** | Mesoderm | 0.0458 | * | Yes | up |
| ***COMMD3*** | Stem Cell | < 0.0001 | **** | Yes | down |
| ***CRABP2*** | Stem Cell | 0.0701 | ns | No | N/A |
| ***DDX4*** | Germ Cell | 0.2226 | ns | No | N/A |
| ***DES*** | Mesoderm | 0.4887 | ns | No | N/A |
| ***DNMT3B*** | Stem Cell | 0.1261 | ns | No | N/A |
| ***EBAF*** | Stem Cell | 0.2974 | ns | No | N/A |
| ***EOMES*** | Trophoblast | 0.0847 | ns | No | N/A |
| ***FGF4*** | Stem Cell | 0.0071 | ** | Yes | up |
| ***FGF5*** | Stem Cell | < 0.0001 | **** | Yes | down |
| ***FLT1*** | Endoderm | 0.0399 | * | Yes | up |
| ***FN1*** | Endoderm | 0.0102 | * | Yes | down |
| ***FOXA2*** | Endoderm | 0.0007 | *** | Yes | up |
| ***FOXD3*** | Stem Cell | 0.1689 | ns | No | N/A |
| ***GABRB3*** | Stem Cell | 0.1389 | ns | No | N/A |
| ***GAL*** | Stem Cell | < 0.0001 | **** | Yes | down |
| ***GATA4*** | Extra-embryonic endoderm | 0.0381 | * | Yes | up |
| ***GATA6*** | Stem Cell | 0.0149 | * | Yes | down |
| ***GBX2*** | Stem Cell | 0.2504 | ns | No | N/A |
| ***GCG*** | Endoderm | 0.1061 | ns | No | N/A |
| ***GCM1*** | Trophoblast | 0.544 | ns | No | N/A |
| ***GDF3*** | Stem Cell | 0.6708 | ns | No | N/A |
| ***GFAP*** | Ectoderm | 0.958 | ns | No | N/A |
| ***GRB7*** | Stem Cell | < 0.0001 | **** | Yes | up |
| ***HBB*** | Mesoderm | 0.3963 | ns | No | N/A |
| ***HBZ*** | Mesoderm | 0.7261 | ns | No | N/A |
| ***HLXB9*** | Ectoderm | 0.9289 | ns | No | N/A |
| **GENE** | **GROUP** | p value | p value summary | Significantly different? (p < 0.05) | Up/Down-regulation |
| ***IAPP*** | Endoderm | 0.8005 | ns | No | N/A |
| ***IFITM1*** | Stem Cell | 0.3156 | ns | No | N/A |
| ***IFITM2*** | Stem Cell | 0.0003 | *** | Yes | down |
| ***IL6ST*** | Stem Cell | < 0.0001 | **** | Yes | down |
| ***IMP2*** | Stem Cell | 0.0663 | ns | No | N/A |
| ***INS*** | Endoderm | N/A | N/A | N/A | N/A |
| ***IPF1*** | Endoderm | 0.0977 | ns | No | N/A |
| ***ISL1*** | Ectoderm | 0.9787 | ns | No | N/A |
| ***KIT*** | Stem Cell | 0.0004 | *** | Yes | up |
| ***KRT1*** | Trophoblast | 0.2711 | ns | No | N/A |
| ***LAMA1*** | Endoderm | 0.3691 | ns | No | N/A |
| ***LAMB1*** | Endoderm | 0.003 | ** | Yes | down |
| ***LAMC1*** | Endoderm | < 0.0001 | **** | Yes | down |
| ***LEFTB*** | Stem Cell | 0.0006 | *** | Yes | down |
| ***LIFR*** | Stem Cell | 0.2465 | ns | No | N/A |
| ***LIN28*** | Stem Cell | 0.0001 | *** | Yes | up |
| ***MYF5*** | Mesoderm | < 0.0001 | **** | Yes | up |
| ***MYOD1*** | Mesoderm | 0.1546 | ns | No | N/A |
| ***NANOG*** | Stem Cell | 0.9183 | ns | No | N/A |
| ***NES*** | Ectoderm | 0.1288 | ns | No | N/A |
| ***NEUROD1*** | Ectoderm | 0.0021 | ** | Yes | up |
| ***NODAL*** | Stem Cell | 0.0281 | * | Yes | down |
| ***NOG*** | Stem Cell | 0.3395 | ns | No | N/A |
| ***NR5A2*** | Stem Cell | 0.0002 | *** | Yes | down |
| ***NR6A1*** | Stem Cell | < 0.0001 | **** | Yes | up |
| ***OLIG2*** | Ectoderm | 0.0097 | ** | Yes | up |
| ***PAX4*** | Endoderm | 0.9466 | ns | No | N/A |
| ***PAX6*** | Ectoderm | < 0.0001 | **** | Yes | up |
| ***PECAM1*** | Mesoderm | 0.0205 | * | Yes | down |
| ***PODXL*** | Stem Cell | 0.6773 | ns | No | N/A |
| ***POU5F1*** | Stem Cell | 0.2213 | ns | No | N/A |
| ***PTEN*** | Stem Cell | 0.2213 | ns | No | N/A |
| ***PTF1A*** | Endoderm | 0.0573 | ns | No | N/A |
| ***REST*** | Stem Cell | 0.0364 | * | Yes | up |
| ***RUNX2*** | Mesoderm | < 0.0001 | **** | Yes | down |
| ***SEMA3A*** | Stem Cell | < 0.0001 | **** | Yes | down |
| ***SERPINA1*** | Endoderm | 0.0628 | ns | No | N/A |
| ***SFRP2*** | Stem Cell | 0.0001 | *** | Yes | down |
| **GENE** | **GROUP** | p value | p value summary | Significantly different? (p < 0.05) | Up/Down-regulation |
| ***SOX17*** | Endoderm | 0.0156 | * | Yes | up |
| ***SOX2*** | Stem Cell | < 0.0001 | **** | Yes | up |
| ***SST*** | Endoderm | 0.1108 | ns | No | N/A |
| ***SYCP3*** | Germ Cell | 0.2134 | ns | No | N/A |
| ***SYP*** | Ectoderm | 0.0009 | *** | Yes | up |
| ***T*** | Mesoderm | 0.7201 | ns | No | N/A |
| ***TAT*** | Endoderm | 0.1565 | ns | No | N/A |
| ***TDGF1*** | Stem Cell | 0.4997 | ns | No | N/A |
| ***TERT*** | Stem Cell | 0.3452 | ns | No | N/A |
| ***TFCP2L1*** | Stem Cell | 0.4302 | ns | No | N/A |
| ***TH*** | Ectoderm | 0.0549 | ns | No | N/A |
| ***UTF1*** | Stem Cell | 0.2132 | ns | No | N/A |
| ***WT1*** | Mesoderm | 0.0024 | ** | Yes | down |
| ***XIST*** | Stem Cell | 0.1468 | ns | No | N/A |
| ***ZFP42*** | Stem Cell | 0.0064 | ** | Yes | up |
