# Supplementary material for: A Combination of Culture Conditions and Gene Expression Analysis Can Be Used to Investigate and Predict hES Cell Differentiation Potential towards Male Gonadal Cells
Source: PLoS One. 2015 Dec 2;10(12):e0144029. doi: 10.1371/journal.pone.0144029 (PMC4667967; doi:10.1371/journal.pone.0144029)
Supplement: S8 Table — A list of gene names and abbreviations can be found in S6 Table. (DOC) [file pone.0144029.s013.doc]

| Bonferroni's multiple comparisons test | Mean Diff. | 95% CI of diff. | Significant? | Summary | Adjusted p Value | Up/Down |
| --- | --- | --- | --- | --- | --- | --- |
| ***ACTC*** | | | | | | |
| 207 hff vs. 207 sus | -0.8551 | -1.667 to -0.04351 | Yes | * | 0.035 | up |
| 207 hff vs. 401 hff | -0.996 | -1.808 to -0.1843 | Yes | * | 0.0113 | up |
| 207 sus vs. 401 sus | -2.912 |  |  |  |  | up |
| 360 hff vs. 360 sus | -1.253 | -2.064 to -0.4410 | Yes | ** | 0.0017 | up |
| 360 hff vs. 401 hff | -1.249 | -2.061 to -0.4377 | Yes | ** | 0.0017 | up |
| 360 sus vs. 401 sus | -2.768 |  |  |  |  | up |
| 401 hff vs. 401 sus | -2.771 |  |  |  |  | up |
| ***AFP*** | | | | | | |
| 207 hff vs. 207 sus | 9.919 | 5.361 to 14.48 | Yes | **** | < 0.0001 | down |
| 207 sus vs. 360 sus | -10.98 | -15.54 to -6.423 | Yes | **** | < 0.0001 | up |
| 360 sus vs. 401 sus | 12.02 |  |  |  |  | down |
| 401 hff vs. 401 sus | 12.02 |  |  |  |  | down |
| ***BRIX*** | | | | | | |
| 207 hff vs. 207 sus | 0.8894 | 0.2950 to 1.484 | Yes | ** | 0.0022 | down |
| 360 hff vs. 360 sus | 0.6169 | 0.02250 to 1.211 | Yes | * | 0.0389 | down |
| ***CD9*** | | | | | | |
| 207 sus vs. 360 sus | -0.7079 | -1.369 to -0.04679 | Yes | * | 0.0312 | up |
| 207 sus vs. 401 sus | -0.8695 |  |  |  |  | up |
| 401 hff vs. 401 sus | -0.7136 |  |  |  |  | up |
| ***CDH5*** | | | | | | |
| 207 sus vs. 401 sus | -6.136 |  |  |  |  | up |
| ***CDX2*** | | | | | | |
| 207 hff vs. 207 sus | 7.181 | 0.5775 to 13.79 | Yes | * | 0.028 | down |
| ***CGB*** | | | | | | |
| 360 sus vs. 401 sus | 1.499 |  |  |  |  | down |
| 401 hff vs. 401 sus | 1.97 |  |  |  |  | down |
| Bonferroni's multiple comparisons test | Mean Diff. | 95% CI of diff. | Significant? | Summary | Adjusted p Value | Up/Down |
| ***COL1A1*** | | | | | | |
| 207 hff vs. 207 sus | -6.262 | -6.960 to -5.563 | Yes | **** | < 0.0001 | up |
| 207 hff vs. 360 hff | 0.7816 | 0.08316 to 1.480 | Yes | * | 0.0228 | down |
| 207 hff vs. 401 hff | 0.8448 | 0.1463 to 1.543 | Yes | * | 0.0127 | down |
| 207 sus vs. 360 sus | -1.115 | -1.814 to -0.4167 | Yes | ** | 0.0012 | up |
| 207 sus vs. 401 sus | -0.9587 |  |  |  |  | up |
| 360 hff vs. 360 sus | -8.158 | -8.857 to -7.460 | Yes | **** | < 0.0001 | up |
| 401 hff vs. 401 sus | -8.065 |  |  |  |  | up |
| ***COL2A1*** | | | | | | |
| 207 hff vs. 207 sus | 1.637 | 0.4858 to 2.788 | Yes | ** | 0.0034 | down |
| 207 sus vs. 360 sus | -1.39 | -2.542 to -0.2393 | Yes | * | 0.0128 | up |
| 207 sus vs. 401 sus | -2.822 |  |  |  |  | up |
| 360 hff vs. 360 sus | 1.23 | 0.07895 to 2.381 | Yes | * | 0.0317 | down |
| 360 sus vs. 401 sus | -1.432 |  |  |  |  | up |
| 401 hff vs. 401 sus | -1.207 |  |  |  |  | up |
| ***COMMD3*** | | | | | | |
| 207 hff vs. 207 sus | -1.723 | -2.713 to -0.7333 | Yes | *** | 0.0005 | up |
| 360 hff vs. 360 sus | -1.782 | -2.772 to -0.7917 | Yes | *** | 0.0004 | up |
| 401 hff vs. 401 sus | -2.316 |  |  |  |  | up |
| ***CRABP2*** | | | | | | |
| 207 sus vs. 401 sus | 1.132 |  |  |  |  | down |
| 360 sus vs. 401 sus | 1.121 |  |  |  |  | down |
| 401 hff vs. 401 sus | 1.244 |  |  |  |  | down |
| ***DES*** | | | | | | |
| 207 hff vs. 360 hff | -1.74 | -2.726 to -0.7538 | Yes | *** | 0.0005 | up |
| 207 sus vs. 360 sus | -1.994 | -2.980 to -1.008 | Yes | *** | 0.0001 | up |
| 207 sus vs. 401 sus | -1.846 |  |  |  |  | up |
| Bonferroni's multiple comparisons test | Mean Diff. | 95% CI of diff. | Significant? | Summary | Adjusted p Value | Up/Down |
| ***DNMT3B*** | | | | | | |
| 207 hff vs. 360 hff | -0.7975 | -1.376 to -0.2188 | Yes | ** | 0.0044 | up |
| 207 sus vs. 360 sus | -0.869 | -1.448 to -0.2903 | Yes | ** | 0.0021 | up |
| ***EBAF*** | | | | | | |
| 207 hff vs. 207 sus | -1.317 | -1.999 to -0.6350 | Yes | *** | 0.0002 | up |
| 207 hff vs. 360 hff | -0.7433 | -1.425 to -0.06144 | Yes | * | 0.0275 | up |
| 207 hff vs. 401 hff | -1.388 | -2.070 to -0.7064 | Yes | *** | 0.0001 | up |
| 207 sus vs. 360 sus | 1.103 | 0.4211 to 1.785 | Yes | ** | 0.0011 | down |
| 207 sus vs. 401 sus | -3.83 |  |  |  |  | up |
| 360 sus vs. 401 sus | -4.933 |  |  |  |  | up |
| 401 hff vs. 401 sus | -3.758 |  |  |  |  | up |
| ***EOMES*** | | | | | | |
| 207 hff vs. 207 sus | 2.747 | 1.052 to 4.442 | Yes | ** | 0.0011 | down |
| 207 hff vs. 401 hff | 2.323 | 0.6277 to 4.018 | Yes | ** | 0.0046 | down |
| ***FGF4*** | | | | | | |
| 207 hff vs. 360 hff | -1.082 | -1.935 to -0.2282 | Yes | ** | 0.0088 | up |
| 207 hff vs. 401 hff | -1.423 | -2.277 to -0.5697 | Yes | *** | 0.0008 | up |
| 207 sus vs. 401 sus | -1.036 |  |  |  |  | up |
| 360 hff vs. 360 sus | 1.054 | 0.2005 to 1.908 | Yes | * | 0.0108 | down |
| 401 hff vs. 401 sus | 0.9046 |  |  |  |  | down |
| ***FGF5*** | | | | | | |
| 207 hff vs. 207 sus | -12.56 | -15.53 to -9.585 | Yes | **** | < 0.0001 | up |
| 360 hff vs. 360 sus | -13.11 | -16.08 to -10.13 | Yes | **** | < 0.0001 | up |
| 401 hff vs. 401 sus | -14.39 |  |  |  |  | up |
|  |  |  |  |  |  |  |
|  |  |  |  |  |  |  |
|  |  |  |  |  |  |  |
| Bonferroni's multiple comparisons test | Mean Diff. | 95% CI of diff. | Significant? | Summary | Adjusted p Value | Up/Down |
| ***FOXA2*** | | | | | | |
| 207 hff vs. 207 sus | 7.502 | 4.390 to 10.61 | Yes | **** | < 0.0001 | down |
| 207 hff vs. 401 hff | 6.019 | 2.908 to 9.131 | Yes | *** | 0.0002 | down |
| 360 hff vs. 360 sus | 5.778 | 2.667 to 8.889 | Yes | *** | 0.0003 | down |
| 360 hff vs. 401 hff | 3.725 | 0.6132 to 6.836 | Yes | * | 0.0137 | down |
| ***GABRB3*** | | | | | | |
| 207 hff vs. 207 sus | 1.053 | 0.6039 to 1.501 | Yes | **** | < 0.0001 | down |
| 207 sus vs. 360 sus | -1.134 | -1.583 to -0.6854 | Yes | **** | < 0.0001 | up |
| 207 sus vs. 401 sus | -1.271 |  |  |  |  | up |
| ***GAL*** | | | | | | |
| 207 hff vs. 207 sus | -2.572 | -3.166 to -1.979 | Yes | **** | < 0.0001 | up |
| 207 hff vs. 401 hff | -1.173 | -1.767 to -0.5795 | Yes | *** | 0.0002 | up |
| 207 sus vs. 401 sus | -2.432 |  |  |  |  | up |
| 360 hff vs. 360 sus | -2.921 | -3.515 to -2.328 | Yes | **** | < 0.0001 | up |
| 360 sus vs. 401 sus | -2.455 |  |  |  |  | up |
| 401 hff vs. 401 sus | -3.832 |  |  |  |  | up |
| ***GATA4*** | | | | | | |
| 207 hff vs. 207 sus | 3.831 | 0.8129 to 6.849 | Yes | ** | 0.0087 | down |
| 207 sus vs. 360 sus | -3.495 | -6.513 to -0.4774 | Yes | * | 0.0176 | up |
| 360 hff vs. 401 hff | 3.533 | 0.5154 to 6.551 | Yes | * | 0.0163 | down |
| ***GATA6*** | | | | | | |
| 207 hff vs. 401 hff | 2.261 | 0.1114 to 4.411 | Yes | * | 0.0354 | down |
| 207 sus vs. 360 sus | -2.734 | -4.884 to -0.5842 | Yes | ** | 0.0085 | up |
| 360 hff vs. 360 sus | -3.229 | -5.379 to -1.079 | Yes | ** | 0.0021 | up |
| 401 hff vs. 401 sus | -2.697 |  |  |  |  | up |
|  | | | | | | |
|  | | | | | | |
| Bonferroni's multiple comparisons test | Mean Diff. | 95% CI of diff. | Significant? | Summary | Adjusted p Value | Up/Down |
| ***GDF3*** | | | | | | |
| 207 hff vs. 360 hff | -1.536 | -1.810 to -1.262 | Yes | **** | < 0.0001 | up |
| 207 hff vs. 401 hff | -1.282 | -1.556 to -1.009 | Yes | **** | < 0.0001 | up |
| 207 sus vs. 360 sus | -1.829 | -2.103 to -1.555 | Yes | **** | < 0.0001 | up |
| 207 sus vs. 401 sus | -2.195 |  |  |  |  | up |
| 360 hff vs. 360 sus | -0.3252 | -0.5989 to -0.05147 | Yes | * | 0.0145 | up |
| 360 sus vs. 401 sus | -0.3658 |  |  |  |  | up |
| 401 hff vs. 401 sus | -0.9449 |  |  |  |  | up |
| ***GFAP*** | | | | | | |
| 360 sus vs. 401 sus | 1.648 |  |  |  |  | down |
| ***GRB7*** | | | | | | |
| 207 hff vs. 207 sus | 1.216 | 0.7377 to 1.695 | Yes | **** | < 0.0001 | down |
| 360 hff vs. 360 sus | 1.196 | 0.7173 to 1.674 | Yes | **** | < 0.0001 | down |
| 360 sus vs. 401 sus | 0.5103 |  |  |  |  | down |
| 401 hff vs. 401 sus | 1.639 |  |  |  |  | down |
| ***HBZ*** | | | | | | |
| 207 hff vs. 360 hff | -2.257 | -4.371 to -0.1429 | Yes | * | 0.0319 | up |
| ***IFITM1*** | | | | | | |
| 207 hff vs. 360 hff | -0.9801 | -1.401 to -0.5590 | Yes | **** | < 0.0001 | up |
| 207 sus vs. 360 sus | -0.6378 | -1.059 to -0.2167 | Yes | ** | 0.002 | up |
| 207 sus vs. 401 sus | 0.538 |  |  |  |  | down |
| 360 hff vs. 401 hff | 0.5949 | 0.1738 to 1.016 | Yes | ** | 0.0036 | down |
| 360 sus vs. 401 sus | 1.176 |  |  |  |  | down |
| 401 hff vs. 401 sus | 0.905 |  |  |  |  | down |
|  |  |  |  |  |  |  |
|  |  |  |  |  |  |  |
|  |  |  |  |  |  |  |
| Bonferroni's multiple comparisons test | Mean Diff. | 95% CI of diff. | Significant? | Summary | Adjusted p Value | Up/Down |
| ***IFITM2*** | | | | | | |
| 207 hff vs. 207 sus | -1.562 | -2.256 to -0.8686 | Yes | **** | < 0.0001 | up |
| 207 hff vs. 360 hff | -0.734 | -1.428 to -0.04022 | Yes | * | 0.034 | up |
| 207 sus vs. 360 sus | -0.9814 | -1.675 to -0.2876 | Yes | ** | 0.0035 | up |
| 207 sus vs. 401 sus | 1.298 |  |  |  |  | down |
| 360 hff vs. 360 sus | -1.81 | -2.504 to -1.116 | Yes | **** | < 0.0001 | up |
| 360 hff vs. 401 hff | 1.316 | 0.6222 to 2.010 | Yes | *** | 0.0002 | down |
| 360 sus vs. 401 sus | 2.279 |  |  |  |  | down |
| 401 hff vs. 401 sus | -0.8464 |  |  |  |  | up |
| ***IL6ST*** | | | | | | |
| 207 hff vs. 207 sus | -3.001 | -3.843 to -2.158 | Yes | **** | < 0.0001 | up |
| 207 sus vs. 401 sus | -1.26 |  |  |  |  | up |
| 360 hff vs. 360 sus | -3.398 | -4.240 to -2.555 | Yes | **** | < 0.0001 | up |
| 401 hff vs. 401 sus | -4.25 |  |  |  |  | up |
| ***IMP2*** | | | | | | |
| 207 sus vs. 401 sus | -0.5536 |  |  |  |  | up |
| 360 hff vs. 360 sus | -0.5189 | -0.9620 to -0.07576 | Yes | * | 0.0162 | up |
| ***ISL1*** | | | | | | |
| 207 sus vs. 360 sus | -1.365 | -2.453 to -0.2766 | Yes | ** | 0.0095 | up |
| 207 sus vs. 401 sus | 1.68 |  |  |  |  | down |
| 360 sus vs. 401 sus | 3.045 |  |  |  |  | down |
| 401 hff vs. 401 sus | 2.174 |  |  |  |  | down |
|  | | | | | | |
|  | | | | | | |
|  | | | | | | |
|  | | | | | | |
|  | | | | | | |
| Bonferroni's multiple comparisons test | Mean Diff. | 95% CI of diff. | Significant? | Summary | Adjusted p Value | Up/Down |
| ***KIT*** | | | | | | |
| 207 hff vs. 207 sus | 1.609 | 1.162 to 2.056 | Yes | **** | < 0.0001 | down |
| 207 hff vs. 360 hff | -0.7827 | -1.230 to -0.3354 | Yes | *** | 0.0005 | up |
| 207 hff vs. 401 hff | -0.5436 | -0.9910 to -0.09623 | Yes | * | 0.0122 | up |
| 207 sus vs. 360 sus | -1.502 | -1.950 to -1.055 | Yes | **** | < 0.0001 | up |
| 360 hff vs. 360 sus | 0.8894 | 0.4420 to 1.337 | Yes | *** | 0.0002 | down |
| 360 sus vs. 401 sus | 1.482 |  |  |  |  | down |
| 401 hff vs. 401 sus | 2.132 |  |  |  |  | down |
| ***LAMB1*** | | | | | | |
| 360 hff vs. 360 sus | -0.9664 | -1.798 to -0.1352 | Yes | * | 0.0171 | up |
| ***LAMC1*** | | | | | | |
| 207 hff vs. 207 sus | -1.345 | -1.968 to -0.7225 | Yes | **** | < 0.0001 | up |
| 207 sus vs. 360 sus | -1.117 | -1.740 to -0.4945 | Yes | *** | 0.0004 | up |
| 207 sus vs. 401 sus | -0.7013 |  |  |  |  | up |
| 360 hff vs. 360 sus | -2.713 | -3.336 to -2.091 | Yes | **** | < 0.0001 | up |
| 401 hff vs. 401 sus | -2.433 |  |  |  |  | up |
| ***LEFTB*** | | | | | | |
| 207 hff vs. 207 sus | -1.857 | -2.273 to -1.440 | Yes | **** | < 0.0001 | up |
| 207 sus vs. 360 sus | 0.9289 | 0.5126 to 1.345 | Yes | **** | < 0.0001 | down |
| 207 sus vs. 401 sus | -2.688 |  |  |  |  | up |
| 360 hff vs. 360 sus | -0.9277 | -1.344 to -0.5114 | Yes | **** | < 0.0001 | up |
| 360 sus vs. 401 sus | -3.617 |  |  |  |  | up |
| 401 hff vs. 401 sus | -4.638 |  |  |  |  | up |
|  | | | | | | |
|  | | | | | | |
|  | | | | | | |
|  | | | | | | |
| Bonferroni's multiple comparisons test | Mean Diff. | 95% CI of diff. | Significant? | Summary | Adjusted p Value | Up/Down |
| ***LIFR*** | | | | | | |
| 207 hff vs. 207 sus | 4.464 | 0.2661 to 8.663 | Yes | * | 0.0328 | down |
| 207 sus vs. 401 sus | -5.552 |  |  |  |  | up |
| 360 hff vs. 401 hff | 4.265 | 0.06627 to 8.463 | Yes | * | 0.045 | down |
| 401 hff vs. 401 sus | -4.265 |  |  |  |  | up |
| ***LIN28*** | | | | | | |
| 207 hff vs. 207 sus | 0.7856 | 0.2401 to 1.331 | Yes | ** | 0.003 | down |
| 207 sus vs. 360 sus | -0.5732 | -1.119 to -0.02770 | Yes | * | 0.0357 | up |
| 360 hff vs. 360 sus | 0.7365 | 0.1910 to 1.282 | Yes | ** | 0.0052 | down |
| 401 hff vs. 401 sus | 1.031 |  |  |  |  | down |
| ***MYF5*** | | | | | | |
| 207 hff vs. 207 sus | 3.823 | 0.9562 to 6.690 | Yes | ** | 0.0058 | down |
| 360 hff vs. 360 sus | 4.733 | 1.866 to 7.600 | Yes | *** | 0.0009 | down |
| 401 hff vs. 401 sus | 4.916 |  |  |  |  | down |
| ***NANOG*** | | | | | | |
| 207 sus vs. 401 sus | -1.84 |  |  |  |  | up |
| ***NES*** | | | | | | |
| 207 sus vs. 360 sus | 0.901 | 0.1773 to 1.625 | Yes | * | 0.0101 | down |
| 360 sus vs. 401 sus | -0.9962 |  |  |  |  | up |
| ***NEUROD1*** | | | | | | |
| 207 hff vs. 207 sus | 6.238 | 3.328 to 9.147 | Yes | **** | < 0.0001 | down |
| 207 hff vs. 401 hff | 3.733 | 0.8233 to 6.642 | Yes | ** | 0.008 | down |
| 207 sus vs. 401 sus | -3.123 |  |  |  |  | up |
|  | | | | | | |
|  | | | | | | |
|  | | | | | | |
|  | | | | | | |
| Bonferroni's multiple comparisons test | Mean Diff. | 95% CI of diff. | Significant? | Summary | Adjusted p Value | Up/Down |
| ***NODAL*** | | | | | | |
| 207 hff vs. 207 sus | -0.4818 | -0.8993 to -0.06428 | Yes | * | 0.0181 | up |
| 207 hff vs. 401 hff | 0.4385 | 0.02101 to 0.8561 | Yes | * | 0.0358 | down |
| 207 sus vs. 401 sus | -2.501 |  |  |  |  | up |
| 360 sus vs. 401 sus | -2.892 |  |  |  |  | up |
| 401 hff vs. 401 sus | -3.421 |  |  |  |  | up |
| ***NOG*** | | | | | | |
| 360 hff vs. 401 hff | 1.497 | 0.04786 to 2.945 | Yes | * | 0.0401 | down |
| 360 sus vs. 401 sus | 2.097 |  |  |  |  | down |
| ***NR5A2*** | | | | | | |
| 207 hff vs. 207 sus | -0.735 | -1.114 to -0.3557 | Yes | *** | 0.0002 | up |
| 207 hff vs. 401 hff | -0.423 | -0.8022 to -0.04378 | Yes | * | 0.0233 | up |
| 207 sus vs. 360 sus | -0.9367 | -1.316 to -0.5575 | Yes | **** | < 0.0001 | up |
| 207 sus vs. 401 sus | -1.726 |  |  |  |  | up |
| 360 hff vs. 360 sus | -1.545 | -1.924 to -1.165 | Yes | **** | < 0.0001 | up |
| 360 sus vs. 401 sus | -0.7893 |  |  |  |  | up |
| 401 hff vs. 401 sus | -2.038 |  |  |  |  | up |
| ***NR6A1*** | | | | | | |
| 207 hff vs. 207 sus | 1.769 | 1.141 to 2.397 | Yes | **** | < 0.0001 | down |
| 360 hff vs. 360 sus | 1.202 | 0.5739 to 1.830 | Yes | *** | 0.0002 | down |
| 401 hff vs. 401 sus | 1.078 |  |  |  |  | down |
|  | | | | | | |
|  | | | | | | |
|  | | | | | | |
|  | | | | | | |
|  | | | | | | |
|  | | | | | | |
| Bonferroni's multiple comparisons test | Mean Diff. | 95% CI of diff. | Significant? | Summary | Adjusted p Value | Up/Down |
| ***PAX6*** | | | | | | |
| 207 hff vs. 207 sus | 3.864 | 2.361 to 5.368 | Yes | **** | < 0.0001 | down |
| 207 hff vs. 360 hff | -3.574 | -5.077 to -2.071 | Yes | **** | < 0.0001 | up |
| 207 sus vs. 401 sus | -3.152 |  |  |  |  | up |
| 360 hff vs. 360 sus | 7.023 | 5.520 to 8.526 | Yes | **** | < 0.0001 | down |
| 360 hff vs. 401 hff | 2.546 | 1.043 to 4.049 | Yes | *** | 0.0007 | down |
| 360 sus vs. 401 sus | -2.736 |  |  |  |  | up |
| 401 hff vs. 401 sus | 1.741 |  |  |  |  | up |
| ***PECAM1*** | | | | | | |
| 207 sus vs. 360 sus | -2.914 | -4.425 to -1.403 | Yes | *** | 0.0002 | up |
| 207 sus vs. 401 sus | -2.267 |  |  |  |  | up |
| 360 hff vs. 360 sus | -2.835 | -4.347 to -1.324 | Yes | *** | 0.0003 | up |
| 401 hff vs. 401 sus | -2.152 |  |  |  |  | up |
| ***PODXL*** | | | | | | |
| 207 hff vs. 360 hff | -1.464 | -1.990 to -0.9376 | Yes | **** | < 0.0001 | up |
| 207 hff vs. 401 hff | -0.9079 | -1.434 to -0.3818 | Yes | *** | 0.0006 | up |
| 207 sus vs. 360 sus | -1.323 | -1.849 to -0.7971 | Yes | **** | < 0.0001 | up |
| 360 hff vs. 401 hff | 0.5558 | 0.02962 to 1.082 | Yes | * | 0.0344 | down |
| 360 sus vs. 401 sus | 1.551 |  |  |  |  | down |
| 401 hff vs. 401 sus | 0.7288 |  |  |  |  | down |
| ***POU5F1*** | | | | | | |
| 207 hff vs. 401 hff | -0.6141 | -1.228 to -0.0001627 | Yes | * | 0.0499 | up |
| 207 sus vs. 401 sus | -1.392 |  |  |  |  | up |
| 360 hff vs. 401 hff | -0.7689 | -1.383 to -0.1550 | Yes | ** | 0.0097 | up |
| 360 sus vs. 401 sus | -1.054 |  |  |  |  | up |
|  | | | | | | |
| Bonferroni's multiple comparisons test | Mean Diff. | 95% CI of diff. | Significant? | Summary | Adjusted p Value | Up/Down |
| ***PTEN*** | | | | | | |
| 207 hff vs. 401 hff | 1.431 | 0.3144 to 2.547 | Yes | ** | 0.008 | down |
| 207 sus vs. 401 sus | 2.52 |  |  |  |  | down |
| 360 sus vs. 401 sus | 2.425 |  |  |  |  | down |
| 401 hff vs. 401 sus | 1.128 |  |  |  |  | down |
| ***REST*** | | | | | | |
| 207 hff vs. 207 sus | 0.4585 | 0.1052 to 0.8119 | Yes | ** | 0.0073 | down |
| 207 hff vs. 360 hff | -0.4586 | -0.8120 to -0.1053 | Yes | ** | 0.0072 | up |
| 207 sus vs. 360 sus | -0.7742 | -1.128 to -0.4209 | Yes | **** | < 0.0001 | up |
| 360 sus vs. 401 sus | 0.7545 |  |  |  |  | down |
| 401 hff vs. 401 sus | 0.7594 |  |  |  |  | down |
| ***RUNX2*** | | | | | | |
| 207 hff vs. 207 sus | -4.545 | -5.746 to -3.345 | Yes | **** | < 0.0001 | up |
| 360 hff vs. 360 sus | -4.484 | -5.684 to -3.284 | Yes | **** | < 0.0001 | up |
| 401 hff vs. 401 sus | -4.65 |  |  |  |  | up |
| ***SEMA3A*** | | | | | | |
| 207 hff vs. 207 sus | -2.021 | -2.626 to -1.415 | Yes | **** | < 0.0001 | up |
| 207 hff vs. 401 hff | -0.6694 | -1.275 to -0.06414 | Yes | * | 0.0248 | up |
| 207 sus vs. 401 sus | -1.797 |  |  |  |  | up |
| 360 hff vs. 360 sus | -2.035 | -2.640 to -1.429 | Yes | **** | < 0.0001 | up |
| 360 sus vs. 401 sus | -1.273 |  |  |  |  | up |
| 401 hff vs. 401 sus | -3.148 |  |  |  |  | up |
| ***SERPINA1*** | | | | | | |
| 207 sus vs. 360 sus | -5.687 | -10.89 to -0.4892 | Yes | * | 0.0268 | up |
| 360 sus vs. 401 sus | 5.522 |  |  |  |  | down |
| 401 hff vs. 401 sus | 6.303 |  |  |  |  | down |
|  | | | | | | |
| Bonferroni's multiple comparisons test | Mean Diff. | 95% CI of diff. | Significant? | Summary | Adjusted p Value | Up/Down |
| ***SFRP2*** | | | | | | |
| 207 sus vs. 401 sus | -0.8742 |  |  |  |  | up |
| 360 hff vs. 360 sus | -0.7757 | -1.369 to -0.1822 | Yes | ** | 0.0069 | up |
| 360 sus vs. 401 sus | -0.8194 |  |  |  |  | up |
| 401 hff vs. 401 sus | -1.469 |  |  |  |  | up |
| ***SOX17*** | | | | | | |
| 207 hff vs. 207 sus | 7.201 | 4.086 to 10.32 | Yes | **** | < 0.0001 | down |
| 207 hff vs. 401 hff | 5.215 | 2.100 to 8.329 | Yes | *** | 0.0008 | down |
| 207 sus vs. 360 sus | -3.682 | -6.796 to -0.5675 | Yes | * | 0.0151 | up |
| 360 hff vs. 401 hff | 3.762 | 0.6476 to 6.876 | Yes | * | 0.0128 | down |
| ***SOX2*** | | | | | | |
| 207 hff vs. 207 sus | 1.296 | 0.3178 to 2.275 | Yes | ** | 0.0061 | down |
| 360 hff vs. 360 sus | 1.3 | 0.3212 to 2.278 | Yes | ** | 0.006 | down |
| 401 hff vs. 401 sus | 1.844 |  |  |  |  | down |
| ***SST*** | | | | | | |
| 207 hff vs. 207 sus | -1.262 | -2.199 to -0.3257 | Yes | ** | 0.0053 | up |
| 207 hff vs. 360 hff | -2.172 | -3.108 to -1.235 | Yes | **** | < 0.0001 | up |
| 207 hff vs. 401 hff | -2.329 | -3.266 to -1.393 | Yes | **** | < 0.0001 | up |
| 207 sus vs. 360 sus | -1.861 | -2.797 to -0.9241 | Yes | *** | 0.0002 | up |
| 207 sus vs. 401 sus | -4.45 |  |  |  |  | up |
| 360 hff vs. 360 sus | -0.951 | -1.887 to -0.01449 | Yes | * | 0.0451 | up |
| 360 sus vs. 401 sus | -2.59 |  |  |  |  | up |
| 401 hff vs. 401 sus | -3.383 |  |  |  |  | up |
| ***SYCP3*** | | | | | | |
| 207 hff vs. 360 hff | -2.017 | -3.302 to -0.7315 | Yes | ** | 0.0014 | up |
| 207 hff vs. 401 hff | -1.494 | -2.779 to -0.2083 | Yes | * | 0.0172 | up |
| 207 sus vs. 401 sus | -1.739 |  |  |  |  | up |
| Bonferroni's multiple comparisons test | Mean Diff. | 95% CI of diff. | Significant? | Summary | Adjusted p Value | Up/Down |
| ***SYP*** | | | | | | |
| 207 hff vs. 207 sus | 0.9518 | 0.5289 to 1.375 | Yes | **** | < 0.0001 | down |
| 207 hff vs. 360 hff | -1.645 | -2.068 to -1.222 | Yes | **** | < 0.0001 | up |
| 207 hff vs. 401 hff | -1.021 | -1.444 to -0.5979 | Yes | **** | < 0.0001 | up |
| 207 sus vs. 360 sus | -0.9409 | -1.364 to -0.5180 | Yes | **** | < 0.0001 | up |
| 207 sus vs. 401 sus | -0.6354 |  |  |  |  | up |
| 360 hff vs. 360 sus | 1.656 | 1.233 to 2.079 | Yes | **** | < 0.0001 | down |
| 360 hff vs. 401 hff | 0.6242 | 0.2013 to 1.047 | Yes | ** | 0.0025 | down |
| 401 hff vs. 401 sus | 1.337 |  |  |  |  | down |
| ***T*** | | | | | | |
| 207 hff vs. 207 sus | 2.515 | 0.3738 to 4.657 | Yes | * | 0.0159 | down |
| 207 hff vs. 360 hff | 3.149 | 1.008 to 5.290 | Yes | ** | 0.0025 | down |
| 207 hff vs. 401 hff | 3.571 | 1.430 to 5.713 | Yes | *** | 0.0008 | down |
| 207 sus vs. 401 sus | -3.731 |  |  |  |  | up |
| 360 sus vs. 401 sus | -3.601 |  |  |  |  | up |
| 401 hff vs. 401 sus | -4.787 |  |  |  |  | up |
| ***TDGF1*** | | | | | | |
| 207 hff vs. 207 sus | 0.3265 | 0.07786 to 0.5751 | Yes | ** | 0.0066 | down |
| 207 hff vs. 360 hff | -0.8854 | -1.134 to -0.6368 | Yes | **** | < 0.0001 | up |
| 207 hff vs. 401 hff | -0.5844 | -0.8330 to -0.3358 | Yes | **** | < 0.0001 | up |
| 207 sus vs. 401 sus | -1.41 |  |  |  |  | up |
| 360 hff vs. 401 hff | 0.301 | 0.05239 to 0.5496 | Yes | * | 0.0126 | down |
| 401 hff vs. 401 sus | -0.4989 |  |  |  |  | up |
|  | | | | | | |
|  | | | | | | |
|  | | | | | | |
|  | | | | | | |
| Bonferroni's multiple comparisons test | Mean Diff. | 95% CI of diff. | Significant? | Summary | Adjusted p Value | Up/Down |
| ***TERT*** | | | | | | |
| 207 hff vs. 360 hff | -0.5001 | -0.8715 to -0.1288 | Yes | ** | 0.0054 | up |
| 207 hff vs. 401 hff | 0.4545 | 0.08312 to 0.8259 | Yes | * | 0.0116 | down |
| 207 sus vs. 360 sus | -0.7125 | -1.084 to -0.3411 | Yes | *** | 0.0002 | up |
| 360 hff vs. 401 hff | 0.9546 | 0.5833 to 1.326 | Yes | **** | < 0.0001 | down |
| 360 sus vs. 401 sus | 0.7741 |  |  |  |  | down |
| ***TFCP2L1*** | | | | | | |
| 207 hff vs. 401 hff | -0.6292 | -1.235 to -0.02370 | Yes | * | 0.0385 | up |
| 207 sus vs. 360 sus | -1.345 | -1.950 to -0.7390 | Yes | **** | < 0.0001 | up |
| 207 sus vs. 401 sus | -0.6481 |  |  |  |  | up |
| 360 sus vs. 401 sus | 0.6965 |  |  |  |  | down |
| ***UTF1*** | | | | | | |
| 207 hff vs. 360 hff | -1.419 | -2.631 to -0.2059 | Yes | * | 0.0164 | up |
| 207 sus vs. 360 sus | -1.339 | -2.551 to -0.1262 | Yes | * | 0.0251 | up |
| 207 sus vs. 401 sus | 3.05 |  |  |  |  | down |
| 360 hff vs. 401 hff | 2.136 | 0.9239 to 3.349 | Yes | *** | 0.0005 | down |
| 360 sus vs. 401 sus | 4.389 |  |  |  |  | down |
| 401 hff vs. 401 sus | 3.09 |  |  |  |  | down |
| ***WT1*** | | | | | | |
| 207 sus vs. 401 sus | -1.232 |  |  |  |  | up |
| 360 sus vs. 401 sus | -1.59 |  |  |  |  | up |
| 401 hff vs. 401 sus | -2.058 |  |  |  |  | up |
| ***ZFP42*** | | | | | | |
| 207 hff vs. 207 sus | 0.6651 | 0.2178 to 1.112 | Yes | ** | 0.0023 | down |
| 207 sus vs. 360 sus | -0.7179 | -1.165 to -0.2706 | Yes | ** | 0.0012 | up |
| 207 sus vs. 401 sus | -0.5724 |  |  |  |  | up |
